# Seasonal variations in pore water and sediment geochemistry of littoral lake sediments (Asylum Lake, MI, USA)

**DOI:** 10.1186/1467-4866-7-11

**Published:** 2006-12-20

**Authors:** Carla M Koretsky, Johnson R Haas, Douglas Miller, Noah T Ndenga

**Affiliations:** 1 Department of Geosciences, Western Michigan University, Kalamazoo, MI 49008, USA

## Abstract

**Background:**

Seasonal changes in pore water and sediment redox geochemistry have been observed in many near-surface sediments. Such changes have the potential to strongly influence trace metal distribution and thus create seasonal fluctuations in metal mobility and bioavailability.

**Results:**

Seasonal trends in pore water and sediment geochemistry are assessed in the upper 50 cm of littoral kettle lake sediments. Pore waters are always redox stratified, with the least compressed redox stratification observed during fall and the most compressed redox stratification observed during summer. A 2-step sequential sediment extraction yields much more Fe in the first step, targeted at amorphous Fe(III) (hydr)oxides (AEF), then in the second step, which targets Fe(II) monosulfides. Fe extracted in the second step is relatively invariant with depth or season. In contrast, AEF decreases with sediment depth, and is seasonally variable, in agreement with changes in redox stratification inferred from pore water profiles. A 5-step Tessier extraction scheme was used to assess metal association with operationally-defined exchangeable, carbonate, iron and manganese oxide (FMO), organic/sulfide and microwave-digestible residual fractions in cores collected during winter and spring. Distribution of metals in these two seasons is similar. Co, As, Cd, and U concentrations approach detection limits. Fe, Cu and Pb are mostly associated with the organics/sulfides fraction. Cr and Zn are mostly associated with FMO. Mn is primarily associated with carbonates, and Co is nearly equally distributed between the FMO and organics/sulfide fractions.

**Conclusion:**

This study clearly demonstrates that near-surface lake sediment pore water redox stratification and associated solid phase geochemistry vary significantly with season. This has important ramifications for seasonal changes in the bioavailability and mobility of trace elements. Without rate measurements, it is not possible to quantify the contribution of various processes to natural organic matter degradation. However, the pore water and solid phase data suggest that iron reduction and sulfate reduction are the dominant pathways in the upper 50 cm of these sediments.

## 1. Background

Organic-rich freshwater marsh and lake sediments and pore waters show distinct vertical patterns of redox stratification [1-8]. This vertical zonation results, in part, from oxidation of organic matter being coupled to a succession of increasingly less energetically-favorable terminal electron acceptors, e.g. O_2_, NO_3_^-^, Mn(IV), Fe(III), and SO_4_^-2 ^[9]. Typically, zones of Mn and Fe oxide (FMO) enrichment are present just below oxic surface layers. Mn(IV) and Fe(III) form sparingly soluble oxides, which reductively dissolve to produce much more soluble Mn(II) and Fe(II). Thus, accumulation of FMO just below the oxic zone results as Mn(II) and Fe(II) diffuse upwards from deeper, more reduced sediment zones and react with oxygen or nitrate diffusing downward from overlying oxic lake waters or pore waters. In addition to transport via diffusion of dissolved solutes, redox zonation is influenced by transport via advection and due to macrophyte activity, both via bioirrigation and bioturbation, referring to transport of dissolved solutes and particles, respectively.

The relative sizes of redox zones are determined by a complex interplay of factors including temperature, hydrology, lake turnover or mixing and macrophyte and macrofaunal activity, which together influence the balance of organic matter and terminal electron acceptor availability. Seasonal variations in sediment geochemistry, particularly with respect to redox zonation, have been demonstrated in freshwater marsh [10-12] and lake [3-5,13] sediments. More rapid depletion of terminal electron acceptors generally occurs in spring and summer, when labile organic matter is readily available and temperatures are highest. Enhanced reductive dissolution of FMO shallows and thins the suboxic zones. In winter, oxidation of dissolved Fe(II) and Mn(II) leads to reprecipitation of FMO in a thicker, deeper suboxic zone.

This cycle of reductive dissolution and oxidative reprecipitation of FMO is likely to significantly influence the speciation and bioavailability of other metals. Both field and laboratory studies have demonstrated that many trace metals tend to be strongly associated with iron (hydr)oxides [14] or manganese (hydro)oxides [15]. Thus, reductive dissolution of FMO has the potential to mobilize not only Mn and Fe, but also FMO-associated trace metals [3,16-18]. Because metal bioavailability is a function of metal speciation [19-24], seasonal cycles of reductive dissolution and oxidative reprecipitation of FMO may lead to seasonal cycles of metal speciation and biovailability, which could be particularly significant in contaminated environments with elevated metal concentrations. However, it is also possible that reductive dissolution of FMO will not result in the release of metals in dissolved, bioavailable forms.

In sulfidic sediments, trace metal speciation, particularly for chalcophile elements (e.g. Cu, Zn, Pb, Cd) is strongly influenced not only by precipitation of FMO, but also by precipitation of metal sulfides [25-27]. Trace metals may coprecipitate with or adsorb on the surfaces of more abundant Fe sulfide minerals, or, they may form discrete metal sulfide phases [28]. Thus, Mn, Fe or associated trace metals released from reductively dissolved FMO may diffuse downward into more reducing layers of the sediment and become bound in sulfide minerals [29]. In an experimental study, Kerner and Wallmann [30] demonstrated that reductive dissolution of FMO in estuarine sediments released Zn and Cd, which were subsequently precipitated into sulfide phases. Metals thus mobilized from FMO to a sulfide phase may not become any more bioavailable or toxic than they were when associated with FMO. In fact, the ratio of acid volatile sulfides (AVS) to simultaneously extracted metals (SEM) is a widely accepted predictor of sediment toxicity. In laboratory experiments, AVS/SEM ratios higher than one resulted in no mortality of benthic invertebrates [31]. This is presumably because at ratios greater than one, potentially toxic metals are associated with AVS (including both amorphous and crystalline iron sulfides), and thus are not especially bioavailable [28,31,32].

If sulfidic sediments become less reducing, downward diffusion of NO_3_^- ^or O_2_, may oxidize sulfides, potentially releasing associated metals [28,30,32,33]. In lakes with significant seasonal variations in redox stratification, AVS has been shown to undergo measurable cycles of oxidative degradation and reductive precipitation [16,17,34-37]. Thus, seasonal changes in both AVS and FMO have the potentially to significantly influence trace metal speciation and bioavailability in freshwater sediments [17,33-35,37].

The overall goal of the present study is to quantify seasonal changes in the vertical pore water and solid phase geochemistry of shallow littoral sediments in a kettle lake and to determine the influence of such changes on solid phase trace element speciation. Pore water profiles of a suite of redox sensitive species and dissolved nutrients are analyzed, together with trace element distributions in the sediment solid phase inferred from sequential extraction techniques. This study is particularly significant because cm-scale pore water profiles of nutrients and redox sensitive species have been analyzed together with solid phase Fe and trace metal distributions at the same sites during four seasons, resulting in a detailed spatial and temporal depiction of element speciation and distribution.

## 2. Field Site

Asylum Lake is a freshwater kettle lake located within a 274-acre preserve owned by Western Michigan University. The preserve is in an urbanized setting within the city limits of Kalamazoo, MI in southwestern Michigan, USA. The lake is approximately 1100 m long, 270 m wide and has a maximum depth of ~17 m. It is surrounded by gentle slopes to the east and west with steeper slopes to the north and south [38]. Runoff flows from the slightly more elevated western side of the lake toward the eastern side during precipitation events. Wells located less than 35 m from the lakeshore of Asylum Lake, drilled to ~10–15 m depth, show that the underlying sediment is predominantly fine sand to gravel with a few clay lenses [38]. Vegetation at the lakeshore is largely composed of cattails (*Typha sp*) with forested area located within 10 m of the shoreline.

Sediment and pore water samples were collected from a site near the lake shoreline in fall (November 2001), winter (March 2002), spring (June 2002) and summer (September 2002). Samples taken in fall, winter and spring are from an area within 2 m of a location benchmark placed at the southern shoreline of Asylum Lake at the beginning of the study. Sampling during summer was completed at sites ~35 m west of the original benchmark. Air temperatures, monthly precipitation data and surface conditions associated with each sample [39] are shown in Table 1. This region has a significant history of sulfate-rich acid rain precipitation. The National Atmospheric Deposition Program maintains a sampling station in SW Michigan, approximately 25 miles from the study site. Over the past ten years, precipitation at this site had an average pH of 4.49 and an average sulfate concentration of 1.58 g·m^-2^·a^-1 ^[40].

**Table 1:** Detailed descriptions of sample sites.

Sampling Date(s)*	**Monthly Average Air Temperature**^**#**^	**Monthly Precipitation**	**Site Description**
11/29/01 11/30/01	7.8°C	6.7 cm in 11/01	15–20 cm water depth; vegetation present

3/22/02 3/23/02	0.11°C	5.5 cm in 3/02	10–20 cm ice

5/31/02 6/01/02	11.1°C	9.1 cm in 5/02	20–30 cm water depth; water very turbid, grading into fine-grained organic-rich muck; some vegetation present

9/04/02 9/05/02	22.2°C	11.0 cm in 8/02	~35 m distance from original site; 50–75 cm water depth; vegetation present

*Sampling date is date of peeper and core extraction (3–4 weeks after emplacement of peepers).

^#^Monthly air temperature and precipitation averages are from unedited local climatological data provided by NOAA, National Climatic Data Center for the Kalamazoo/Battle Creek International Airport (42°14'N Lat; 85°33'W Lon.; 892 ft elevation).

## 3. Experimental

### 3.1 Pore Water Sampling and Analysis

Pore waters were collected anaerobically at 1–2 cm intervals from the sediment-water interface (SWI) to a depth of 50 cm using pore water diffusion equilibrators ("peepers"' see Ref. [8] for construction details). Prior to deployment in the field, peepers were washed with dilute HCl or HNO_3 _and then kept in a Plexiglas box filled with deionized (DDI) water and degassed using N_2 _for 3 to 4 days. The peepers were then placed in vinyl bags filled with N_2 _and transported to the field, where they were inserted into the sediments and left to equilibrate with the surrounding sediment pore waters for 3–4 weeks. Peepers were emplaced during fall (Nov 2001), winter (Feb 2002), spring (May 2002) and summer (Aug 2002). Upon retrieval (in Dec 2001, Mar 2002, Jun 2002 and Aug 2002) peepers were placed into fresh vinyl bags flushed and filled with N_2_, and were immediately returned to the lab. Pore waters were extracted from each bag using precleaned polypropylene syringes attached to stainless steel needles by piercing both the bag and the dialysis membrane covering each peeper chamber. Each pore water sample was subsequently filtered through a 0.2 μm pore size syringe filter. During the fluid extraction, N_2 _was periodically flushed into the inflated vinyl bag to maintain positive internal pressure through needle holes and avoid leakage of atmospheric O_2 _into the peeper.

All reagents were ACS reagent grade or purer, and all glassware and plasticware was precleaned with trace metal grade nitric acid. Solution pH was immediately measured for each filtered sample, which was then divided into portions for analysis of redox-sensitive species (alkalinity, dissolved Fe^+2^/Fe^+3^, ΣNH_4_^+^, ΣPO_4_^-3^, ΣS^-2^, and SO_4_^-2^). Analyses for ferrous and ferric iron, alkalinity, ammonium, sulfide and phosphate were immediately conducted via UV/Vis spectrophotometry. Samples to be analyzed for sulfate were preserved by acidification using concentrated nitric acid (see Ref. [8] for all spectrophotometric methods). Trace metal concentrations were analyzed using a ThermoElectron PQ ExCell inductively coupled plasma mass spectrometer (ICP-MS). Total dissolved inorganic carbon was analyzed using an OI Analytical 1020A TOC Analyzer.

### 3.2 Sediment Solid Phase Sampling and Analysis

Lake sediment cores were collected from within 1 m of peeper insertion points, within 1–2 days of peeper extraction, using a Russian peat borer (Aquatic Research Instruments Inc.). Core samples were taken in hemispheric sections 0.5 m in length, and were divided on site, under ambient atmospheric conditions, into subsections that were stored in plastic bags. The bags were squeezed shut to remove as much ambient air as possible, sealed, returned to the lab, frozen, and then freeze-dried in a LabConco sediment freeze dryer. Subsamples of freeze-dried sediments were combusted at 550°C for 2 hours for determination of loss on ignition [41].

Sequential sediment extractions were carried out on freeze-dried sediment samples using two different methods [42-44]. The two-step Kostka and Luther extraction scheme is intended to quantify the proportions of amorphous Fe(III) (hydr)oxide phases and iron monosulfides, respectively. Briefly, 10 mL of ascorbic acid reagent (10 g sodium citrate and 10 g sodium bicarbonate in 200 mL N_2_-sparged ≥ 17.5 MΩ water with 4 g ascorbic acid added to give final pH of 8) is added to approximately 1 g of freeze-dried, homogenized sediment from each depth interval. This is mixed on a rotating wheel in a Coy anaerobic chamber (~5% H_2_, 10% CO_2_, 85% N_2_) at 25 rpm for 24 hours, removed from the chamber and centrifuged at 6500 rpm for 30 minutes. The supernatant is filtered through a 0.2 μm syringe filter and analyzed using the ferrozine method for dissolved total Fe. This step releases ascorbate-extractable iron (AEF), which method calibration experiments suggest is primarily comprised of amorphous Fe(III) (hydr)oxides [42]. The remaining sediment is thoroughly washed using ≥ 17.5 MΩ water, and is then extracted for 1 hour using 0.5 M HCl which will liberate acid volatile sulfides (AVS), together with any remaining amorphous Fe(III) oxides [42]. Iron extracted in this step is referred to as HCl-extractable iron (HEF). Filtered supernatants are analyzed immediately using UV/Vis spectrophotometry for dissolved Fe^+3 ^and Fe^+2^.

The five-step Tessier method is intended to assess trace metal partitioning among the following operationally-defined sediment fractions: readily exchangeable, carbonates, iron and manganese oxyhydroxides (FMOs), oxidizable (organics and sulfides) and residuals (primarily silicates). The targeted fractions, sequential extraction reagents and conditions are shown in Table 2. Three modifications are made compared to the original procedure proposed by Tessier et al. [43] to be consistent with methods used by Koretsky et al. [12] and to allow the use of small quantities of sediments. First, only 0.2 g of sediment is used for each extraction step, rather than the 1 g suggested in the original procedure. This should insure sufficient volume of each extracting reagent to remove all of each targeted fraction. Second, only 10 mL of hydroxylamine HCl is used to extract the FMO fraction, compared to 20 mL suggested volume in the original procedure. Due to the small sample size used in this study, this should not limit the extraction of the FMO phase. Lastly, in the residual extraction, microwave digestion is used, rather than the HF/HClO_4 _reagent called for in Step 5 of the original Tessier method. In this study, this step is instead completed using a microwave digestion method modified from the "EPA 3051" method used for rapid soil acid digestion. In PFA-Teflon vessels, 25 mL of 50% HNO_3 _(trace metal grade) was added to the sediment residue remaining after step 4 and heated under controlled pressure and temperature in a MARS5-DS 9015 Microwave Digester. Because HF was not used, a small quantity of refractory material remained after this step. After each extraction step, mixtures are centrifuged at 6500 rpm for 30 minutes and supernatants filtered through 0.2 μm syringe filters. As above, dissolved Fe is analyzed colorimetrically. Concentrated trace metal grade HNO_3 _is added to preserve a second portion of each sample for trace metal analyses using ICP-MS. Between steps, sediment residues are washed repeatedly using ≥ 17.5 MΩ DI water which is carefully drained before addition of the next reagent.

**Table 2:** Procedure used for 5-step Tessier sediment extractions.

**Targeted Fraction**	**Method**
1. Exchangeables	8 mL 1.0 M MgCl_2 _adjusted to pH = 5–7 1 hr at room temperature

2. Carbonates	8 mL 1.0 M sodium acetate adjusted to pH = 5 with acetic acid 5 hrs at room temperature

3. Fe and Mn oxides	10 mL 0.04 M Hydroxylamine HCl in 25% (v/v) acetic acid 6 hrs at 96°C

4. Organics/Sulfides	3 mL 30% H_2_O_2 _adjusted to pH 2 with HNO_3_ 2 hrs at 85°C 3 mL 30% H_2_O_2 _adjusted to pH 2 with HNO_3_ 2 hrs at 85°C Cool. At room temperature add 5 mL of 3.2 N ammonium acetate in 20% (v/v) HNO_3_, dilute to 20 mL and agitate an additional 30 minutes

5. Residual	Microwave digestion [99]

## 4. Results

### 4.1 Pore Waters

Sediment pore waters collected using two sets of peepers during four seasons (total of eight peeper data sets) were analyzed for pH, alkalinity, redox-sensitive dissolved species (dissolved Fe^+2^, Fe^+3^, ΣS^-2^, SO_4_^-2^), dissolved nutrients (ΣNH_4_^+^, ΣPO_4_^-3^) and total dissolved inorganic and organic carbon. Peepers were emplaced in areas separated by not more than ~1.5 m (see above), but were positioned in an attempt to capture maximum heterogeneity (e.g. by placing peepers in regions that were visually sparser or denser with respect to vegetation during fall, spring and summer).

**Figure 1:**
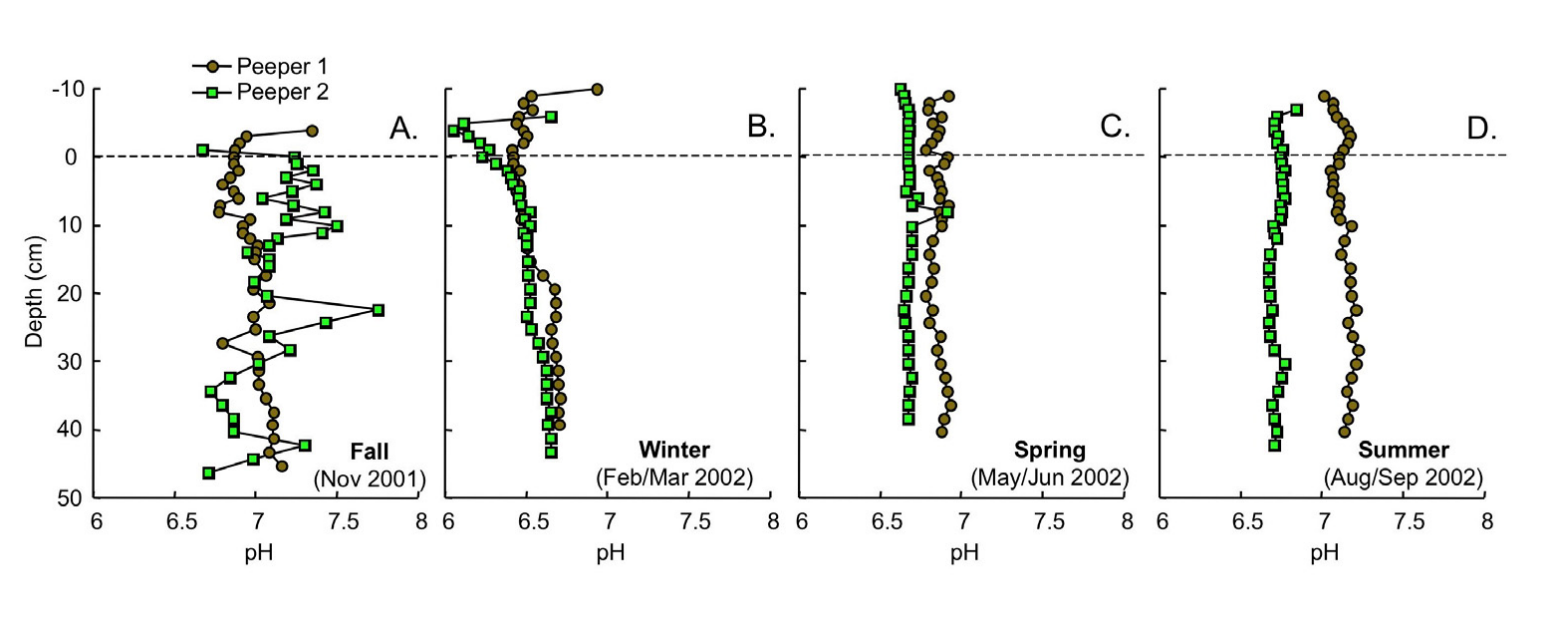
pH as a function of depth measured in replicate peepers in (A) fall (Nov 2001), (B) winter (Feb/Mar 2002), (C) spring (May/Jun 2002) and (D) summer (Aug/Sept 2002). Dashed line indicates the sediment water interface.

During all seasons, pH values are circumneutral with little variation with depth below the sediment water interface (SWI; Fig. 1). Depth-profiles of pH are similar in the replicate peepers, except during summer, when the two profiles are offset by ~0.5 pH. Above the SWI, a decline in pH with depth is sometimes apparent.

**Figure 2:**
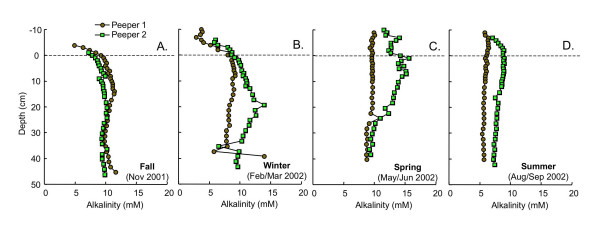
Alkalinity as a function of depth measured in replicate peepers in (A) fall (Nov 2001), (B) winter (Feb/Mar 2002), (C) spring (May/Jun 2002) and (D) summer (Aug/Sept 2002). Dashed line indicates the sediment water interface.

In fall, alkalinity is similar in both peepers, increasing to ~10 mM by a depth of ~8 cm and then remaining essentially constant with depth (Fig. 2A). Replicate profiles are more variable in winter, spring and fall (Figs. 2B–D). In winter, more alkalinity accumulates at shallower depths than in fall (Fig. 2B). In spring, the peeper 1 alkalinity profile is essentially constant with depth (Fig. 2C). In peeper 2, there is an increase in alkalinity from ~11 mM at 10 cm above SWI to ~15 mM at 10 cm depth, followed by a decline to ~10 mM at ~25 cm depth (Fig. 2C). Alkalinities in summer are lower than during other seasons, and vary little with depth (Fig. 2D). Dissolved total inorganic carbon (TIC) profiles (not shown) measured during winter and spring using an OI Analytical TOC Analyzer are in excellent agreement with the total alkalinity values shown in Fig. 2B and 2C, suggesting that the total alkalinity is dominated by the contribution from dissolved carbonate.

**Figure 3:**
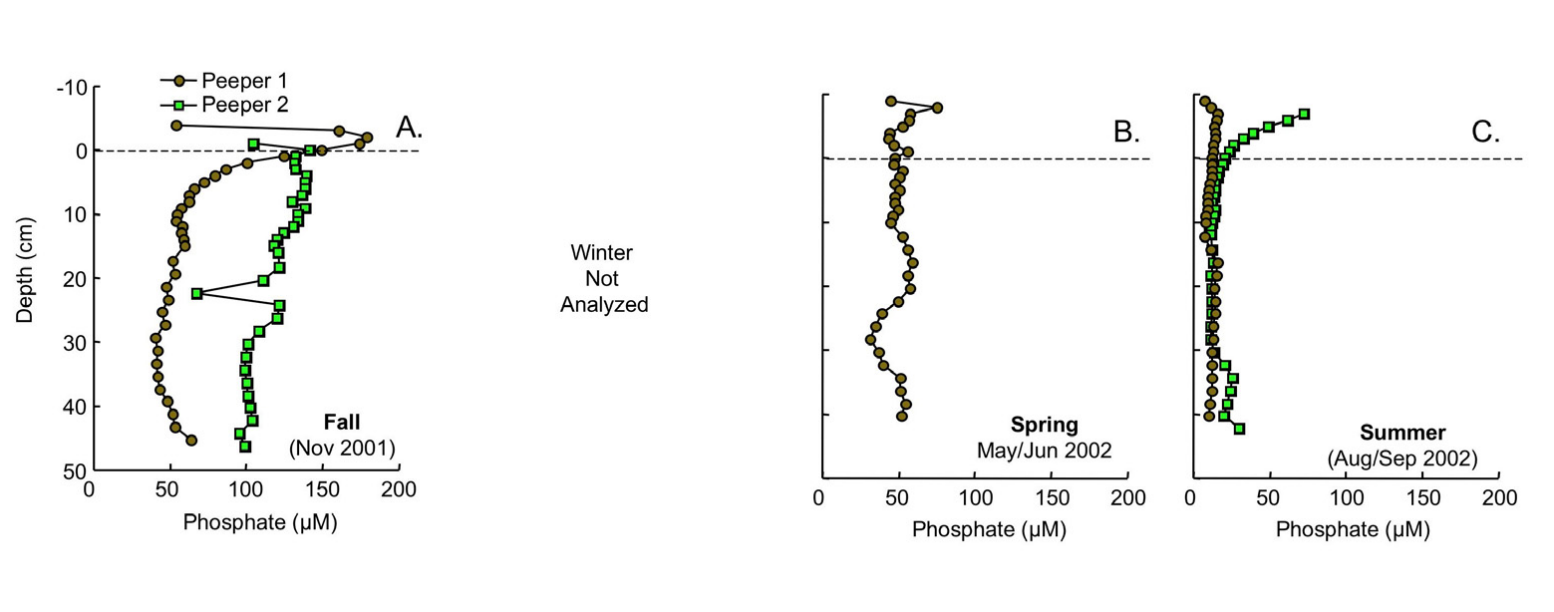
Total dissolved phosphate as a function of depth measured in replicate peepers in (A) fall (Nov 2001), (B) spring (May/Jun 2002) and (C) summer (Aug/Sept 2002). Dashed line indicates the sediment water interface. Phosphate was not measured in winter.

**Figure 4:**
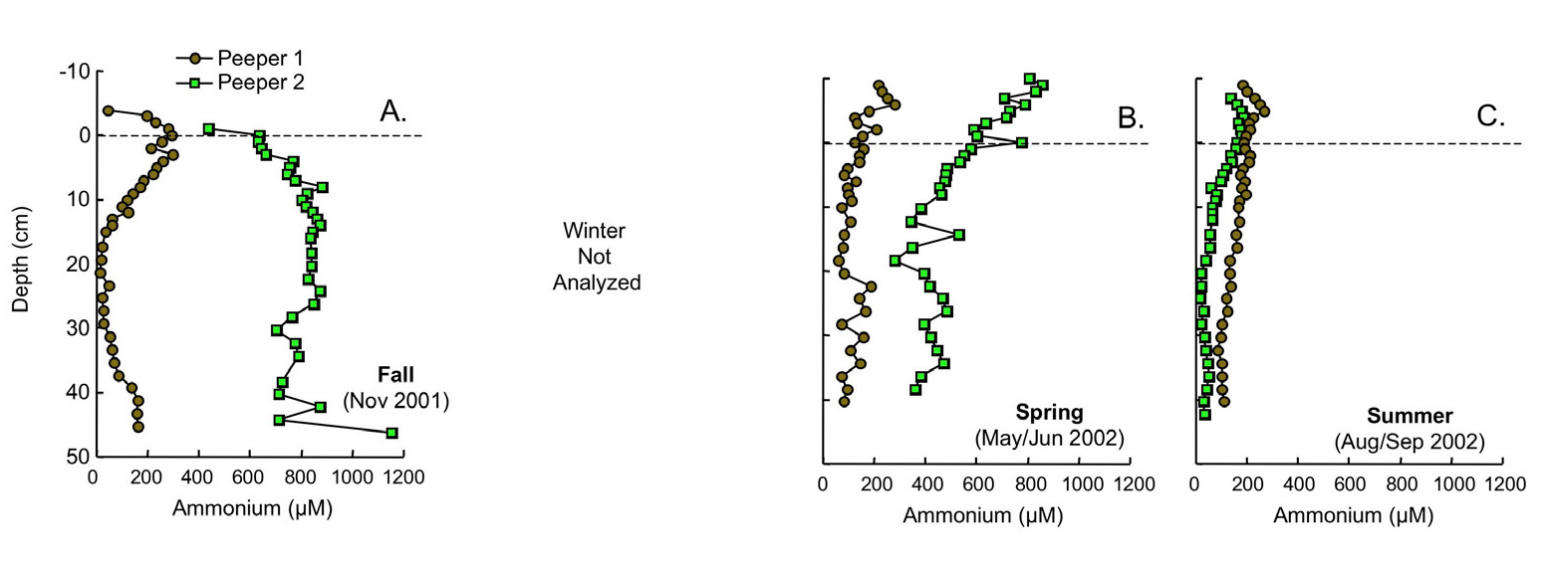
Total dissolved ammonium as a function of depth measured in replicate peepers in (A) fall (Nov 2001), (B) spring (May/Jun 2002) and (C) summer (Aug/Sept 2002). Dashed line indicates the sediment water interface. Ammonium was not measured in winter.

Dissolved nutrient (ammonium and phosphate) profiles are much more spatially and temporally variable than pH or alkalinity (Figs. 3, 4). In fall, peeper 1 shows distinct peaks in dissolved phosphate and ammonium just above the SWI (Fig. 3A, 4A). These sharp peaks are absent in peeper 2, where nutrient concentrations accumulate gradually to much higher levels than in peeper 1 (Fig. 3A, 4A). Nutrients were not analyzed during winter. In spring and summer, dissolved phosphate concentrations are relatively low (~50 μM in spring; ~25 μM in summer) and vary little with depth below the SWI (Fig. 3B,C). Ammonium concentrations in spring, as in fall, are quite variable in the replicate peepers (Fig. 4B). Peeper 2 has concentrations approaching those observed at the high-ammonium site in fall, while peeper 1 has much lower concentrations, similar to levels measured in summer (typically <200 μM). In both summer peepers, dissolved ammonium concentrations are low and vary little with depth (Fig. 4C).

**Figure 5:**
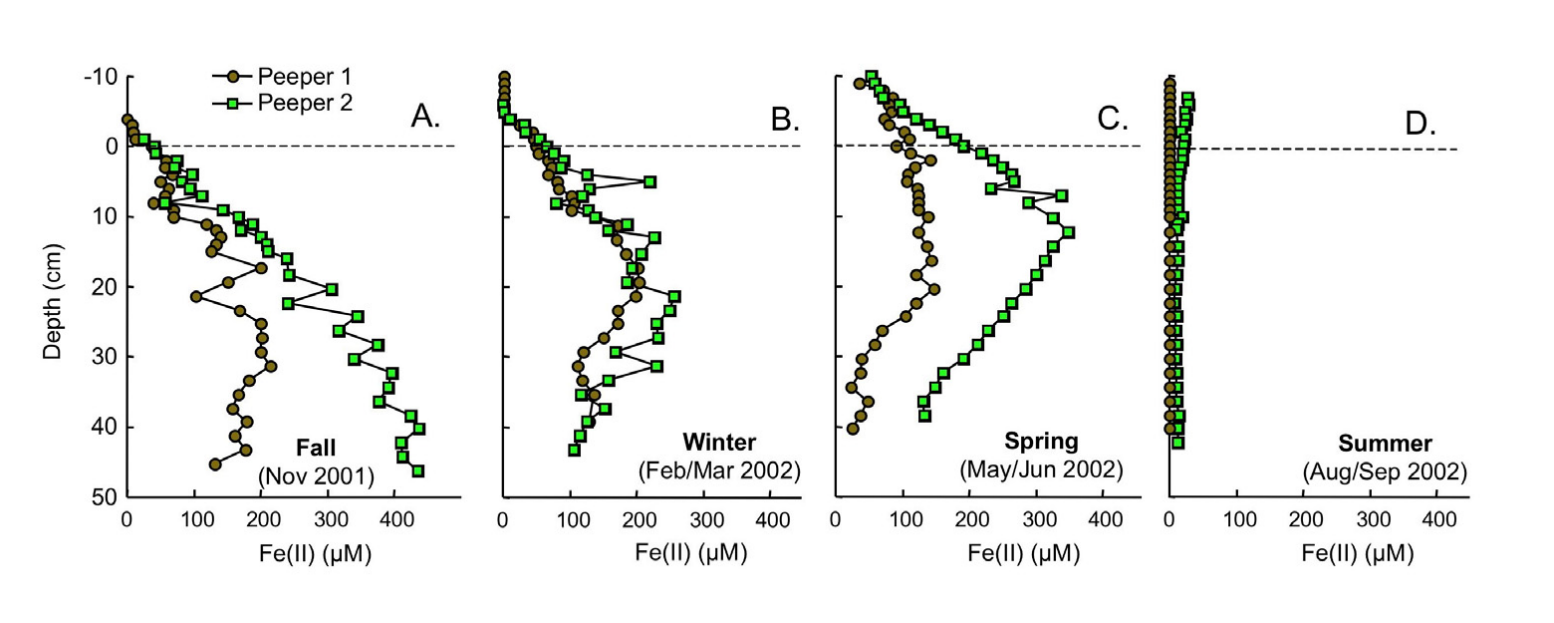
Dissolved Fe(II) as a function of depth measured in replicate peepers in (A) fall (Nov 2001), (B) winter (Feb/Mar 2002), (C) spring (May/Jun 2002) and (D) summer (Aug/Sept 2002). Dashed line indicates the sediment water interface.

Dissolved Fe(II) profiles vary significantly with season (Fig. 5). In fall and spring, Fe(II) profiles are distinct in the two replicates, whereas in winter and summer, replicate dissolved Fe(II) profiles are very similar. In fall, Fe(II) accumulates in the pore waters from near detection limits at the SWI to 100–200 μM at 20 cm depth in peeper 1 and >400 μM at 45 cm depth in peeper 2 (Fig. 5A). In winter, dissolved Fe(II) begins to build up ~4 cm above the SWI, reaching concentrations of ~200 μM by 20 cm depth (Fig. 5B). In spring, Fe(II) concentrations are detectable in samples from 10 cm above the SWI and increase to a maximum of ~350 μM at ~12 cm depth in peeper 1. In peeper 2, Fe(II) concentrations also increase from the uppermost sample, but Fe(II) concentrations are lower (Fig. 5C). In summer, dissolved Fe(II) concentrations are very low (<30 μM) at all depths in both peepers (Fig. 5D).

**Figure 6:**
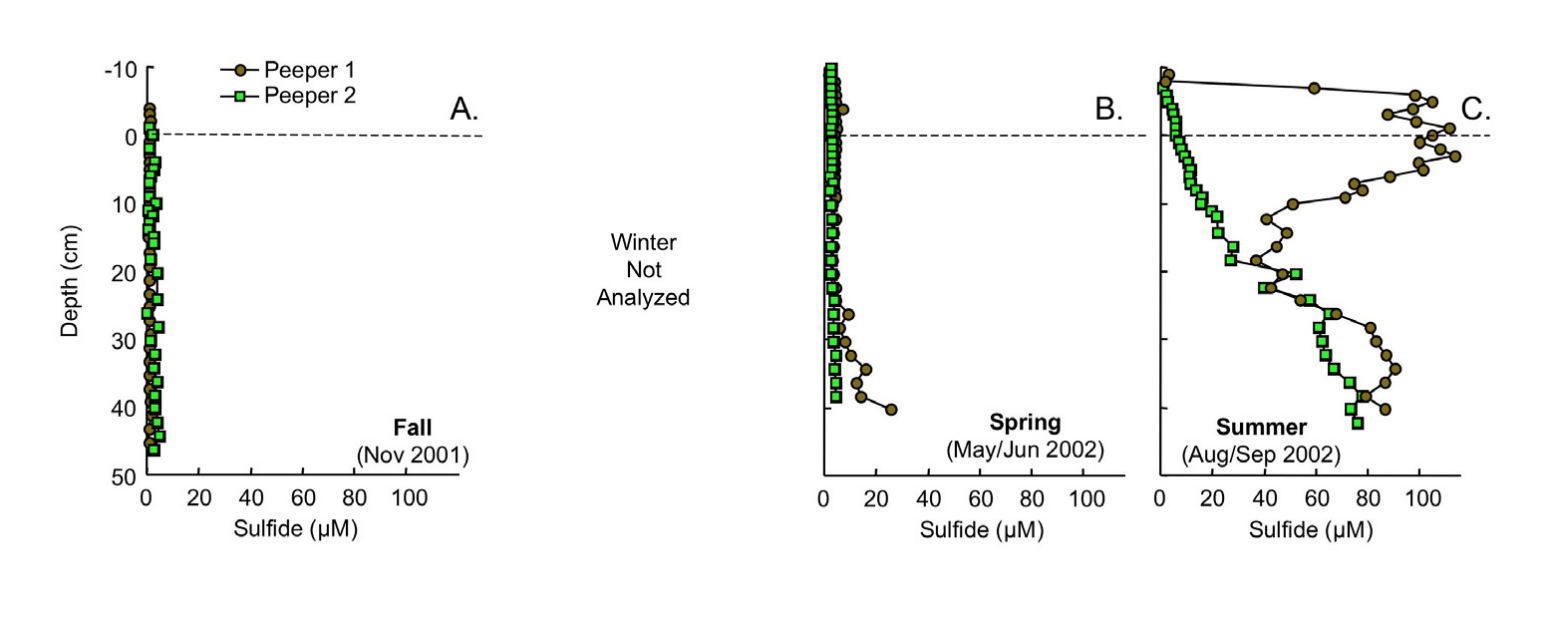
Dissolved sulfide as a function of depth measured in replicate peepers in (A) fall (Nov 2001), (B) spring (May/Jun 2002) and (C) summer (Aug/Sept 2002). Dashed line indicates the sediment water interface. Sulfide was not measured in winter.

Dissolved sulfide concentrations are also seasonally variable (Fig. 6). In fall, sulfide concentrations are always <5 μM (Fig. 6A). During spring, sulfide concentrations are low throughout peeper 2, but begin to increase at a depth of ~25 cm to ~25 μM by 40 cm in the peeper 1 (Fig. 6B). Sulfide concentrations are much higher in summer than in fall or spring. In summer, in peeper 2, sulfide begins to accumulate at least 6 cm above the SWI, steadily increasing to ~75 μM by 40 cm depth. In contrast, in peeper 1 during summer, a broad peak in sulfide occurs, with sulfide levels increasing from <5 μM at 8 cm above the SWI to ~100 μM from 6 cm above to 6 cm below the SWI, then declining to ~35 μM at 20 cm depth. Sulfide then accumulates further with depth, reaching ~90 μM by 40 cm depth (Fig. 6C).

**Figure 7:**
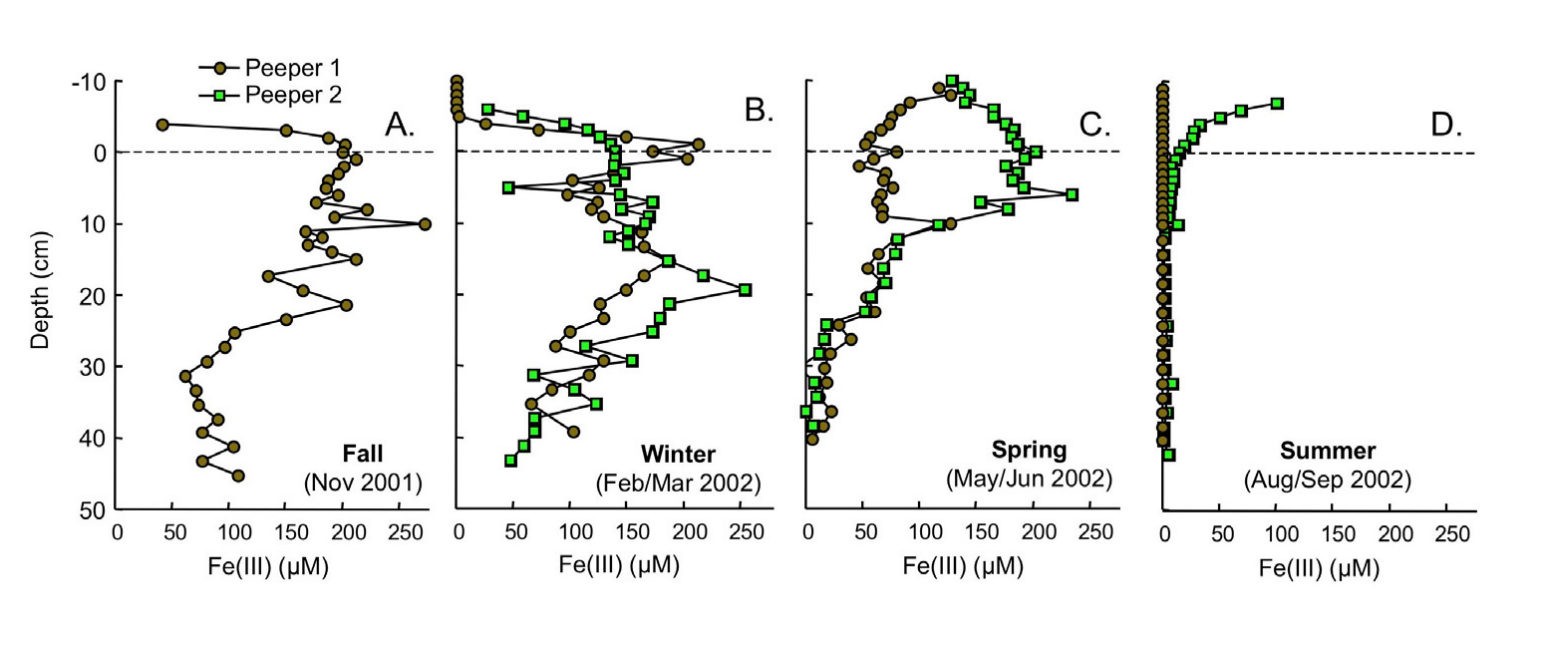
Dissolved Fe(III) as a function of depth measured in replicate peepers in (A) fall (Nov 2001), (B) winter (Feb/Mar 2002), (C) spring (May/Jun 2002) and (D) summer (Aug/Sept 2002). Dashed line indicates the sediment water interface.

Like dissolved Fe(II) and sulfide, pore water Fe(III) varies tremendously with season (Fig. 7). Fe(III) profiles are similar in fall and winter, with Fe(III) accumulating just above the SWI, and then declining with depth (Fig. 7A,B). There is more divergence between replicate Fe(III) depth profiles in spring and summer. In spring, Fe(III) concentrations above 10 cm are much lower in peeper 1. Below 10 cm depths, the profiles are virtually identical (Fig. 7C). In summer, Fe(III) levels are very low (<5 μM) at all analyzed depths in peeper 2. In contrast, in peeper 1, Fe(III) concentrations reach 100 μM at 7 cm above the SWI and decline to < 5 μM by ~10 cm depth (Fig. 7D).

**Figure 8:**
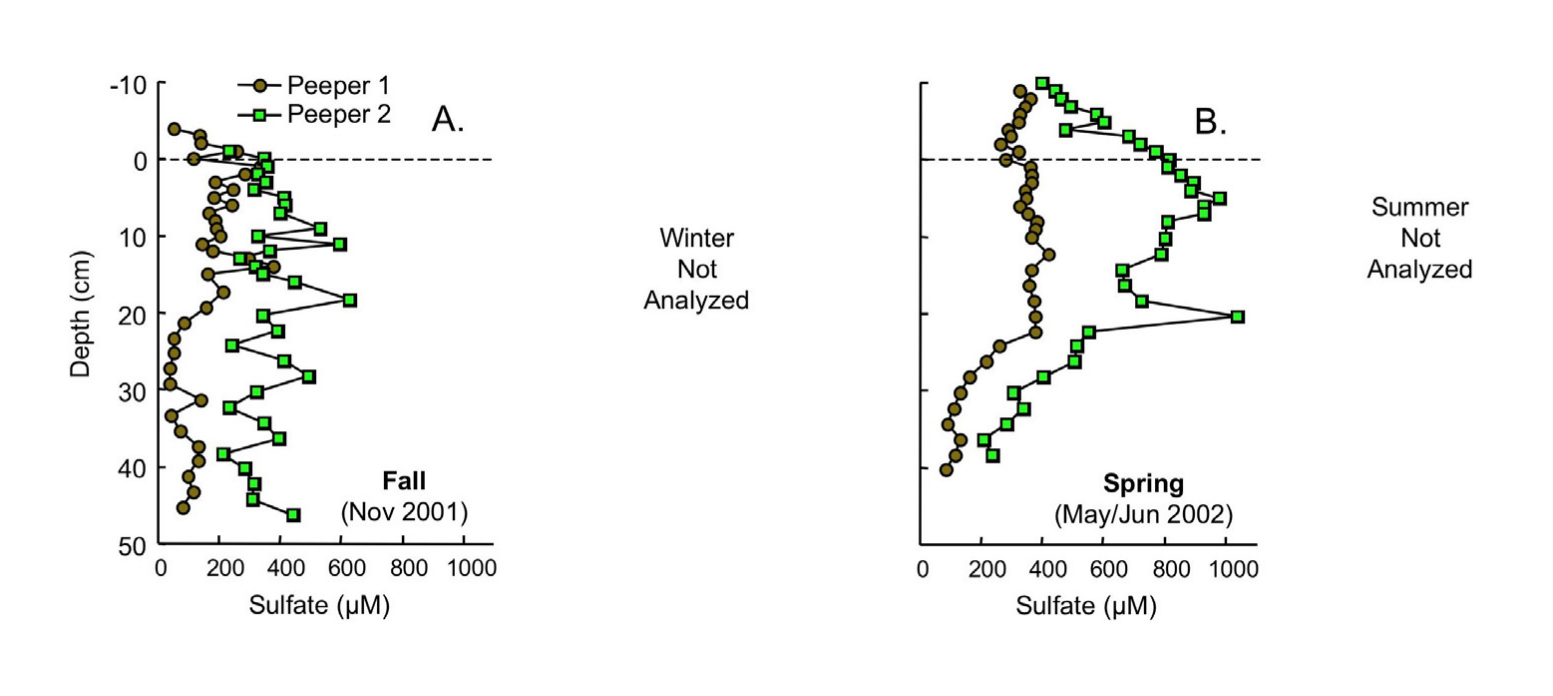
Dissolved sulfate as a function of depth measured in replicate peepers in (A) fall (Nov 2001) and (B) spring (May/Jun 2002). Dashed line indicates the sediment water interface. Sulfate was not measured in winter or summer.

Depth profiles of sulfate were measured in fall and spring only (Fig. 8). Trends of sulfate in the replicate peepers are similar with lower concentrations in peeper 1 during both seasons. In fall, sulfate concentrations in both peepers increase slightly from just above to just below the SWI, and then are relatively constant with depth (Fig. 8A). Sulfate levels are higher in spring than in fall, particularly in peeper 1. Sulfate concentrations in peeper 2 increase from 10 cm above the SWI to ~5 cm depth and then decline slowly with depth. In contrast, sulfate concentrations in peeper 1 vary little from 10 cm above the SWI to ~22 cm depth, and then decline to ~80 μM at 40–45 cm depth (Fig. 8B).

### 4.2 Solid Phase

Three replicate cores at a distance of <1.5 m from each other were collected in fall. Two replicate cores were collected during spring and summer. These cores, like the peepers, were visually located in areas of variable density of vegetation. Because of the difficulty associated with coring through the frozen lake in winter, only one core was taken in winter.

#### 4.2.1 Loss on Ignition (LOI)

**Figure 9:**
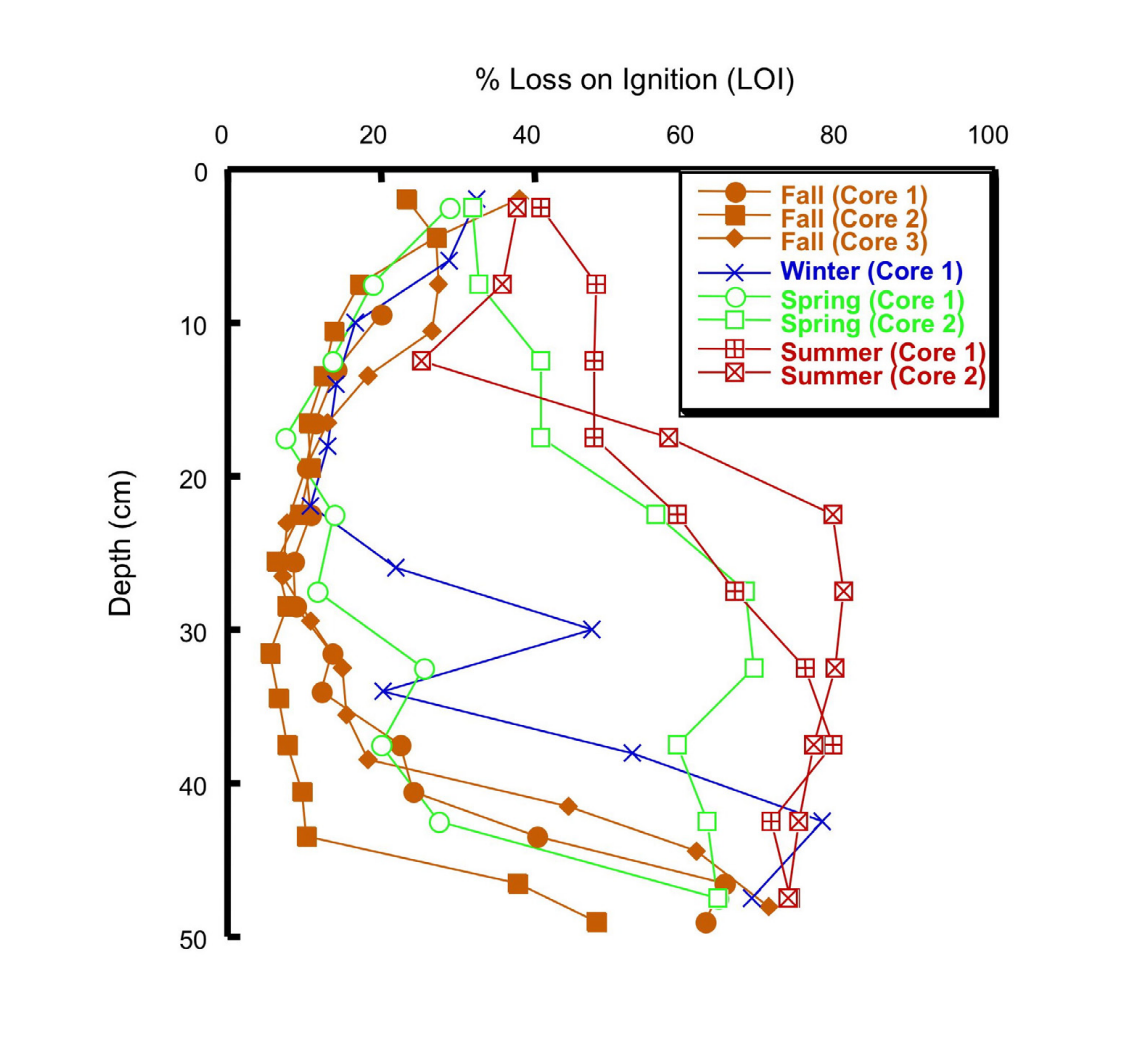
Loss on ignition as a function of depth for cores collected in fall (Dec 2001), winter (Mar 2002), spring (Jun 2002) and summer (Sept 2002).

The organic carbon data from loss on ignition fall into two distinct groups (Fig. 9). LOI in all three fall cores, the winter core and the first spring core have ~30% LOI in the upper portion of the core, decreasing to 10–20% by ~35 cm depth and then increasing dramatically to 50–70% LOI by 50 cm depth. The low LOI zone between 10 and 40 cm depth correlates visually with a distinct color and textural change in the cores. In the higher LOI portions of the core, the sediments are dark brown to black with identifiable roots and organic material, whereas the low LOI portions of the core are finer grained and light grey in color. Both of the summer cores and the second spring core also have LOI values of ~40% at the top of the core, however, in contrast to the first set of cores, in these three cores LOI increases to 60–80% by ~30 cm depth and then varies little from 30–50 cm. No color or texture change was visually apparent in these cores, which were dark brown to black with organic material throughout.

#### 4.2.2 Ascorbate and HCl Extractions

**Figure 10:**
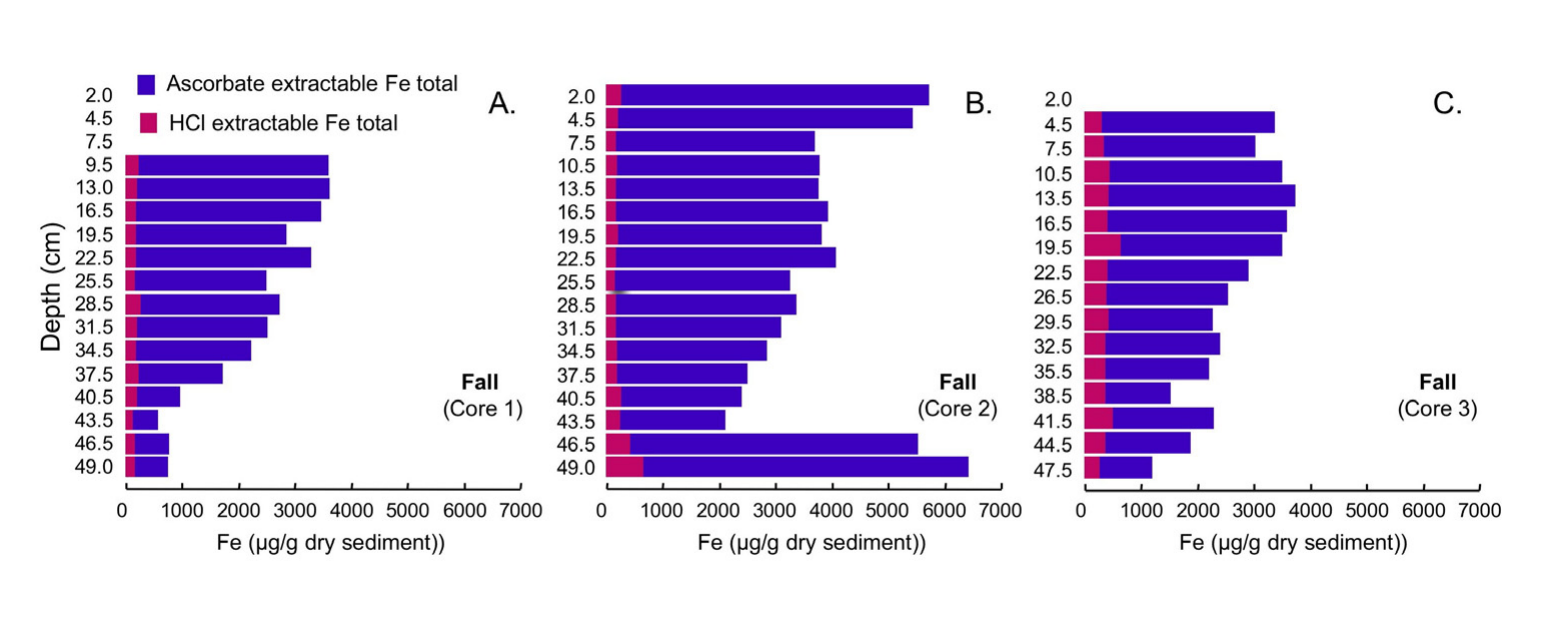
Total Fe concentrations associated with two-step extractions (ascorbate and HCl) completed on triplicate cores collected in fall (Dec 2001).

**Figure 11:**
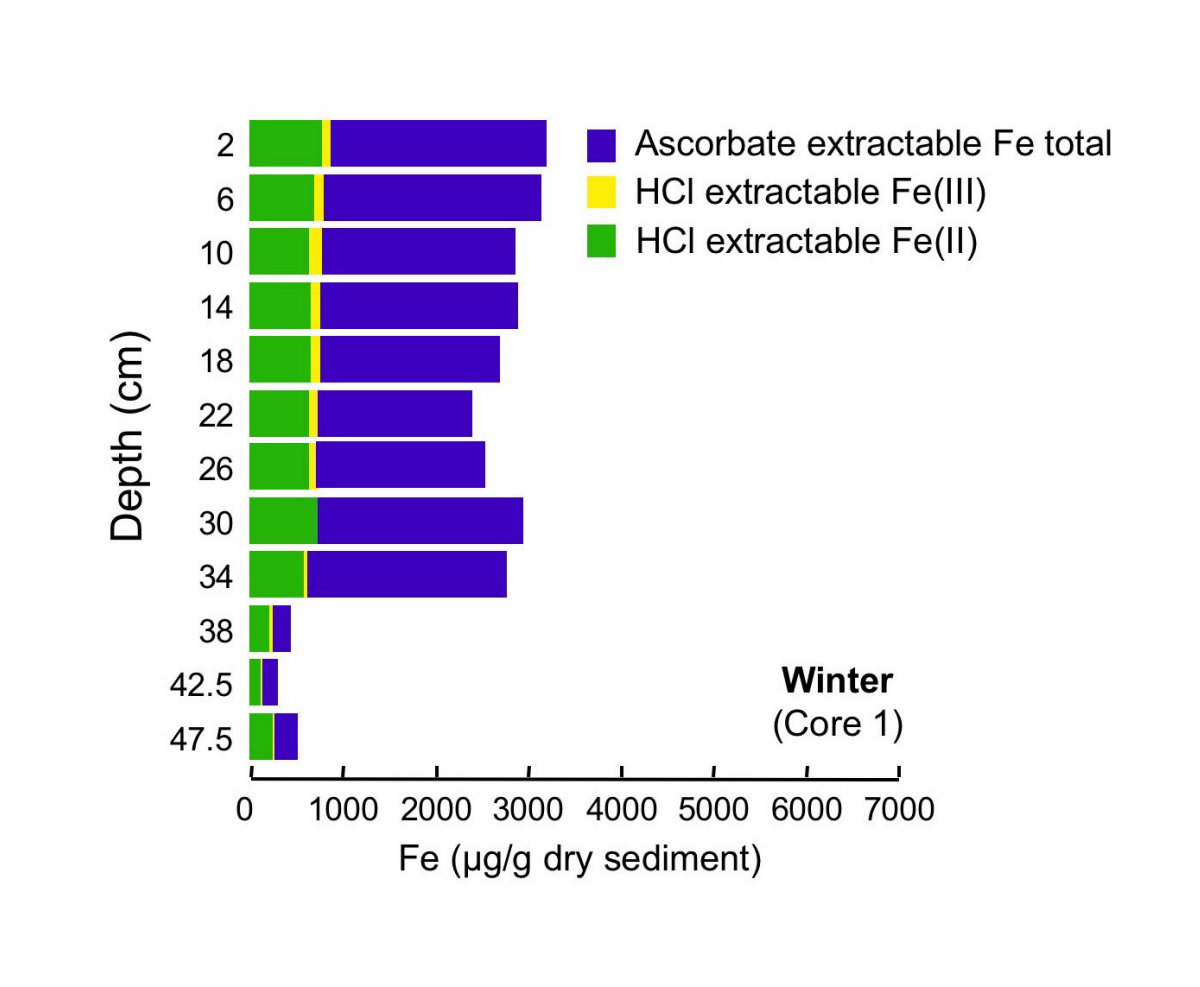
Fe concentrations associated with two-step extractions (ascorbate and HCl) completed on a single core collected in winter (Mar 2002).

**Figure 12:**
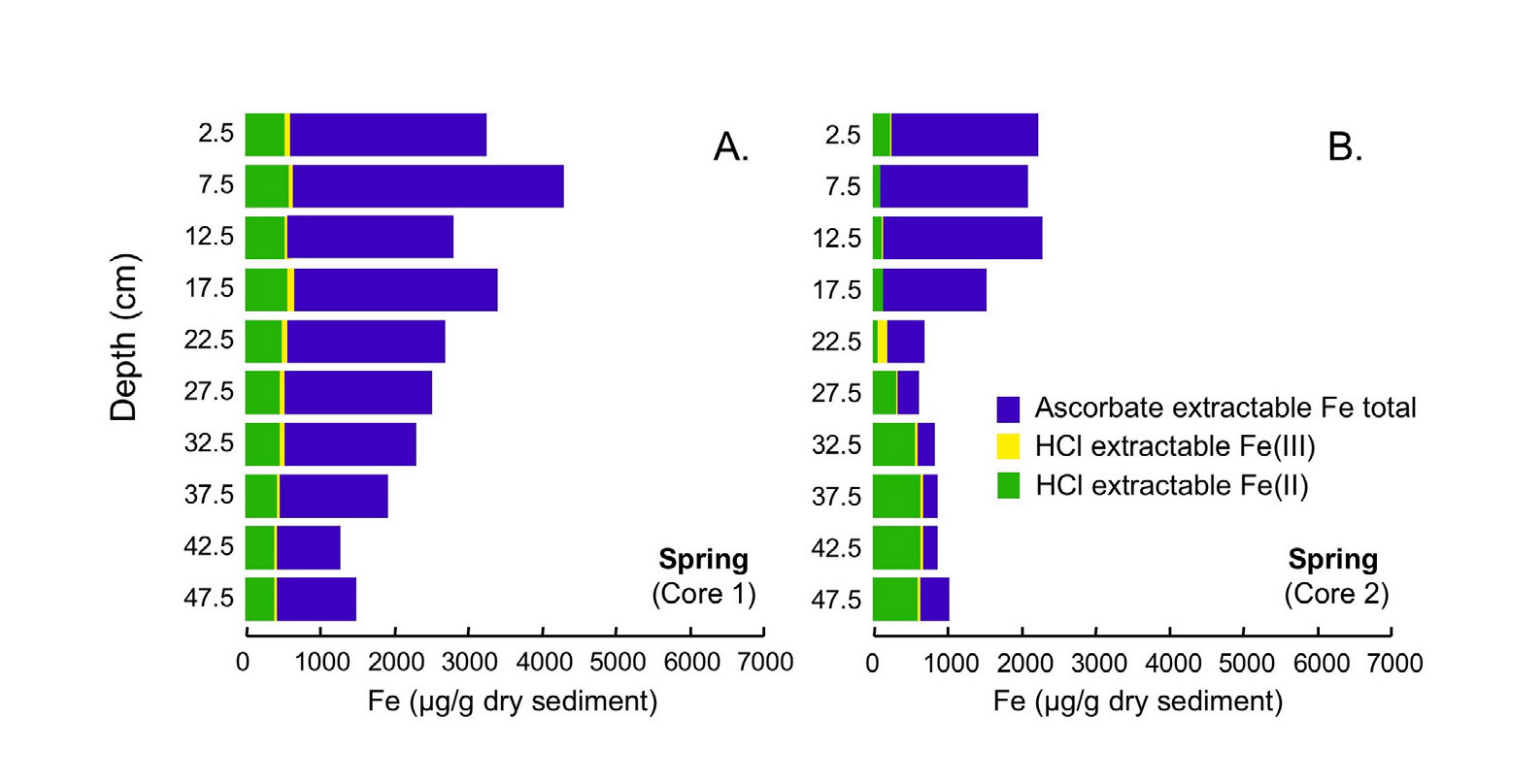
Fe concentrations associated with two-step extractions (ascorbate and HCl) completed on duplicate cores collected in spring (Jun 2002).

**Figure 13:**
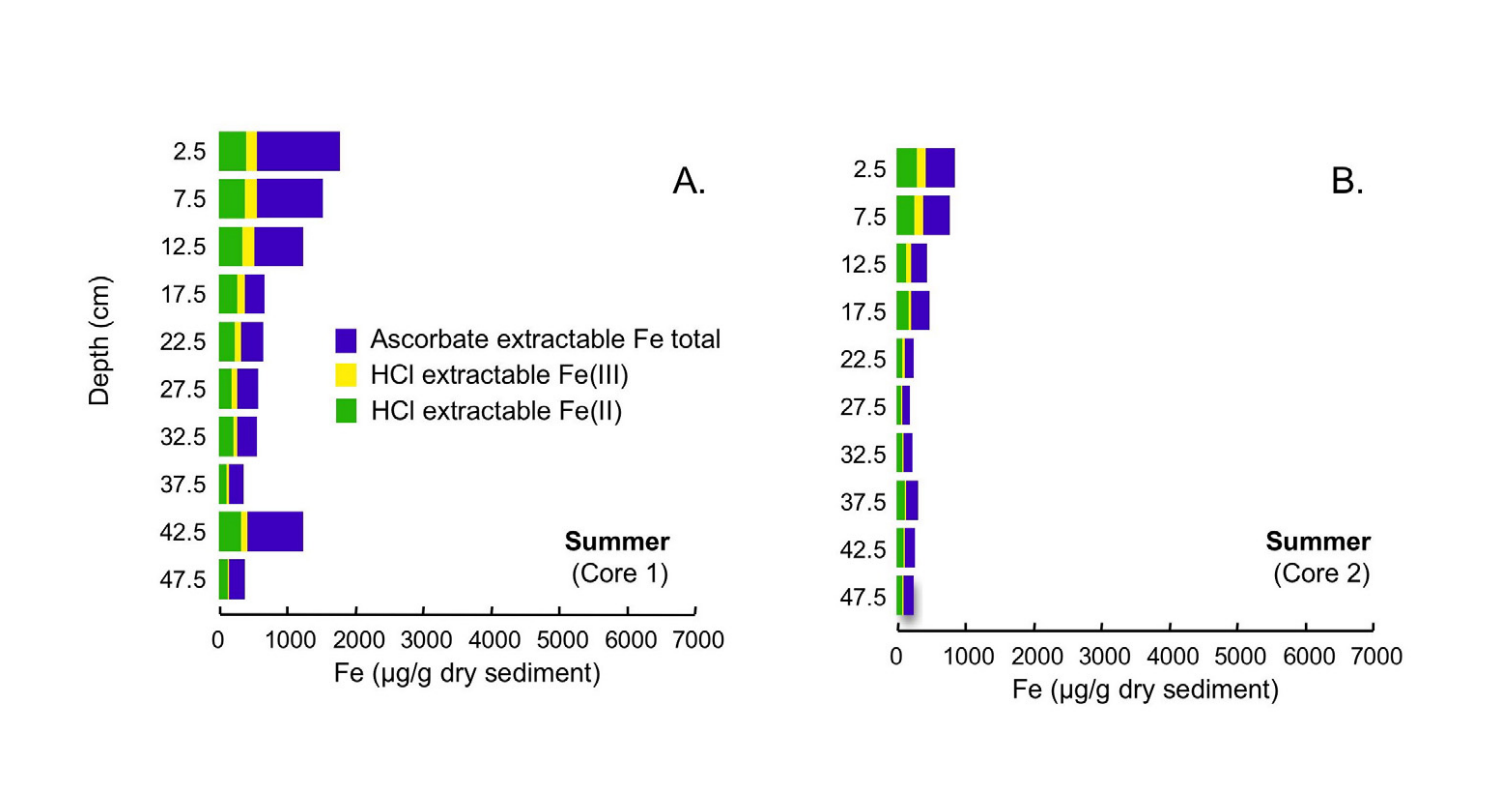
Fe concentrations associated with two-step extractions (ascorbate and HCl) completed on duplicate cores collected in summer (Sept 2002).

The extraction scheme developed by Kostka and Luther [42] to assess reactive solid-phase Fe speciation in marine sediments is used to assess Fe speciation in sediments from cores collected in these sediments during all four seasons. During all four seasons, the majority of the iron at nearly all depths is extracted using ascorbate (Figs. 10, 11, 12, 13). Ascorbate extractable iron (AEF) is typically highest in the uppermost portion of the sediments and decreases with depth. There is also a distinct seasonal variation in AEF. At all sites, the highest concentrations of AEF are measured in fall with declining concentrations in winter and spring and the lowest concentrations measured in summer. Variation of AEF between sites in a single season is typically less than seasonal variations. Concentrations of HCl extractable iron (HEF) are generally <1000 μg/g dry sediment and vary much less than AEF with depth or season. In fall, HEF was analyzed only for total Fe, but in winter, spring and summer, HEF was analyzed for Fe(II)/Fe(III). In nearly all of the extracted samples, the majority of the HEF is comprised of Fe(II).

#### 4.2.3 Tessier Sequential Extractions: Trace Metals

Tessier extractions [43,44] are used to determine the association of Cr, Mn, Fe, Co, Cu, Zn, As, Cd, Pb, and U with four operationally defined sediment fractions: carbonates, iron and manganese oxides (FMO), organics/sulfides, and microwave-digestible residual (primarily silicates) on the core extracted in winter and core 1 extracted in spring. Cobalt is near the detection limits (~0.1 μg/g dry sed) in many of the samples. Cadmium and U is less than 0.1 μg/g dry sed in all samples, and As is typically <2 μg/g dry sed. Arsenic, Cd and U data are not shown. A fifth fraction, readily exchangeable, was extracted, but due to the high ionic strength of the extracting solution and low concentration of the extracted metals could not be analyzed using ICP-MS. This fraction was analyzed via UV/Vis spectrophotometry for Fe, which is found to be negligible (<1% of total extracted Fe at all depths). The microwave-digestible residual fraction dominates Cr, Mn, and Co at all depths (data not shown). In contrast, the microwave-digestible residual fraction typically accounts for less than half of Cu and Zn and less than 25% of Fe and Pb (data not shown). Because the residual fraction is unlikely to be labile, for all metals except Fe, only the distribution of metals in the carbonates, FMO and organics/sulfides fractions are shown here. Fe in all five fractions is shown to facilitate comparison with the Kostka and Luther extractions.

**Figure 14:**
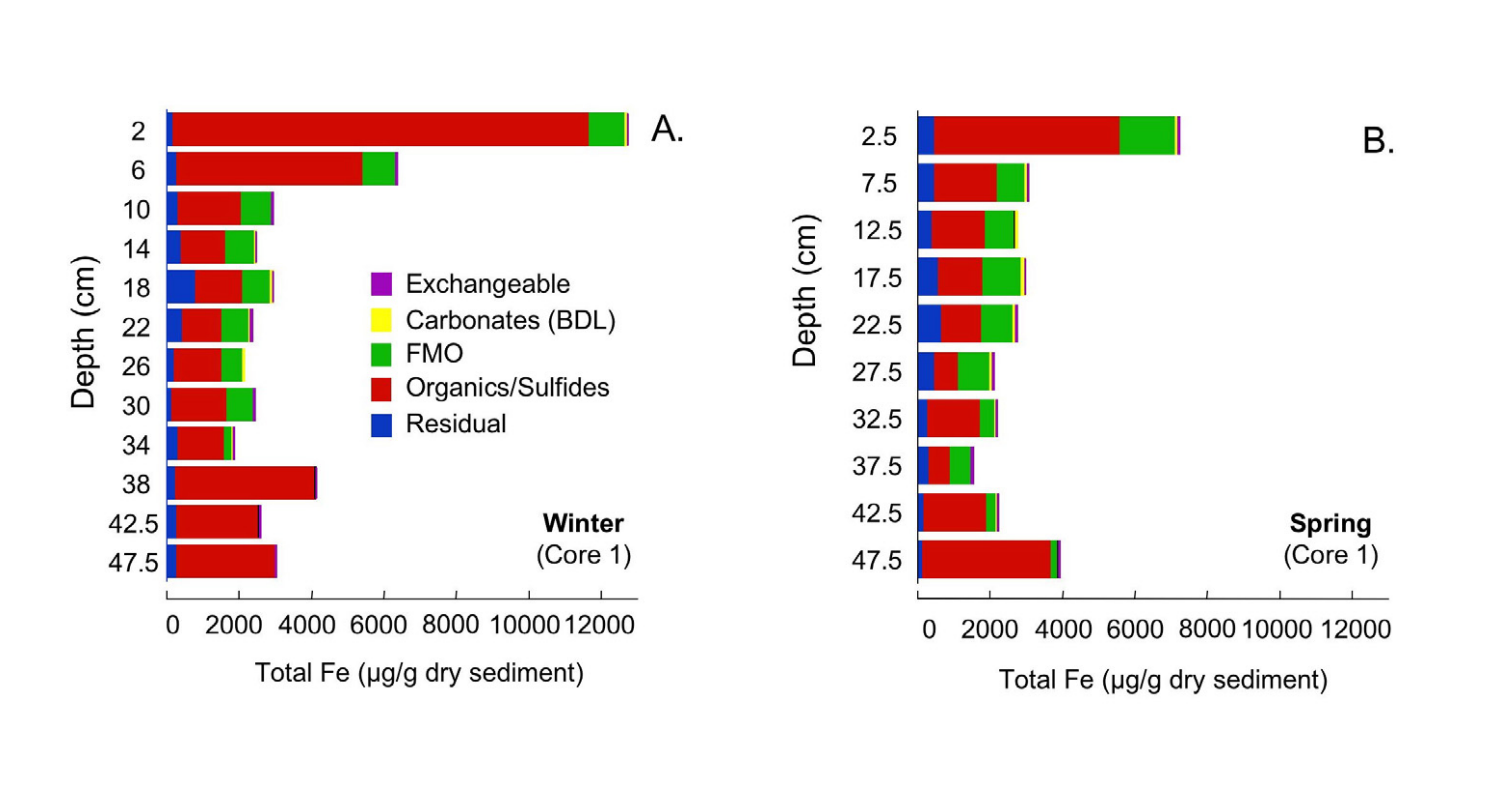
Fe associated with five operationally-defined fractions (exchangeables, carbonates, FMO, organics/sulfides and microwave-digestible residual) for cores collected in (A) winter (Mar 2002) and (B) spring (Jun 2002).

Total extractable Fe reaches >12500 μg/g dry sediment at the top of the sediment column in winter (Fig. 14A). Throughout the core, the majority of Fe is extracted with the organics/sulfides fraction. The amount of organic/sulfide extractable Fe is greatest at the top of the core, intermediate at the bottom of the core, and is least from 10–35 cm depth. Very small percentages, <1% and <2%, respectively, are associated with exchangeable and carbonates fractions. The microwave-digestible residual fraction also accounts for only a small, and relatively constant, amount of extracted Fe. FMO extractable Fe decreases relatively steadily from ~1000 μg/g dry sediment at the top of the core to ~10 μg/g dry sediment at the bottom of the core. With the exception of the upper 2.5 cm, which has much higher Fe concentrations in winter than in spring, the two profiles are similar with respect to both Fe distribution among the five fractions, and overall Fe concentrations (Fig. 14B).

**Figure 15:**
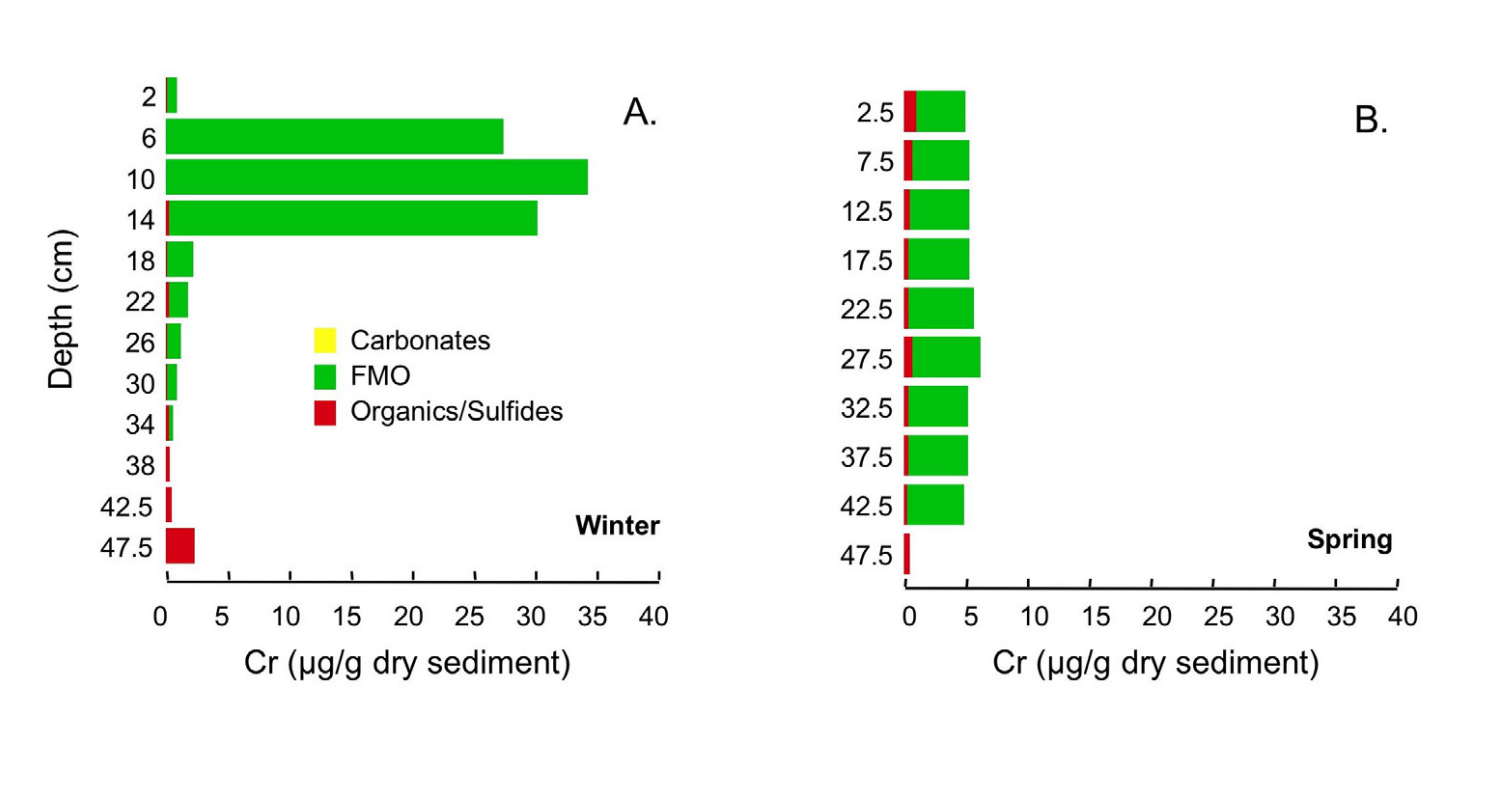
Cr associated with three operationally-defined fractions (carbonates, FMO, and organics/sulfides) for cores collected in (A) winter (Mar 2002) and (B) spring (Jun 2002).

The majority of extractable Cr is associated with the FMO fraction at all depths in both cores (Fig 15). In winter, there is a distinct subsurface maximum in Cr concentration, reaching ~35 μg/g dry sediment, between ~5–15 cm depth (Fig. 15A). In contrast, in spring the extractable Cr concentrations are relatively constant with depth at ~5 μg/g dry sediment (Fig. 15B).

**Figure 16:**
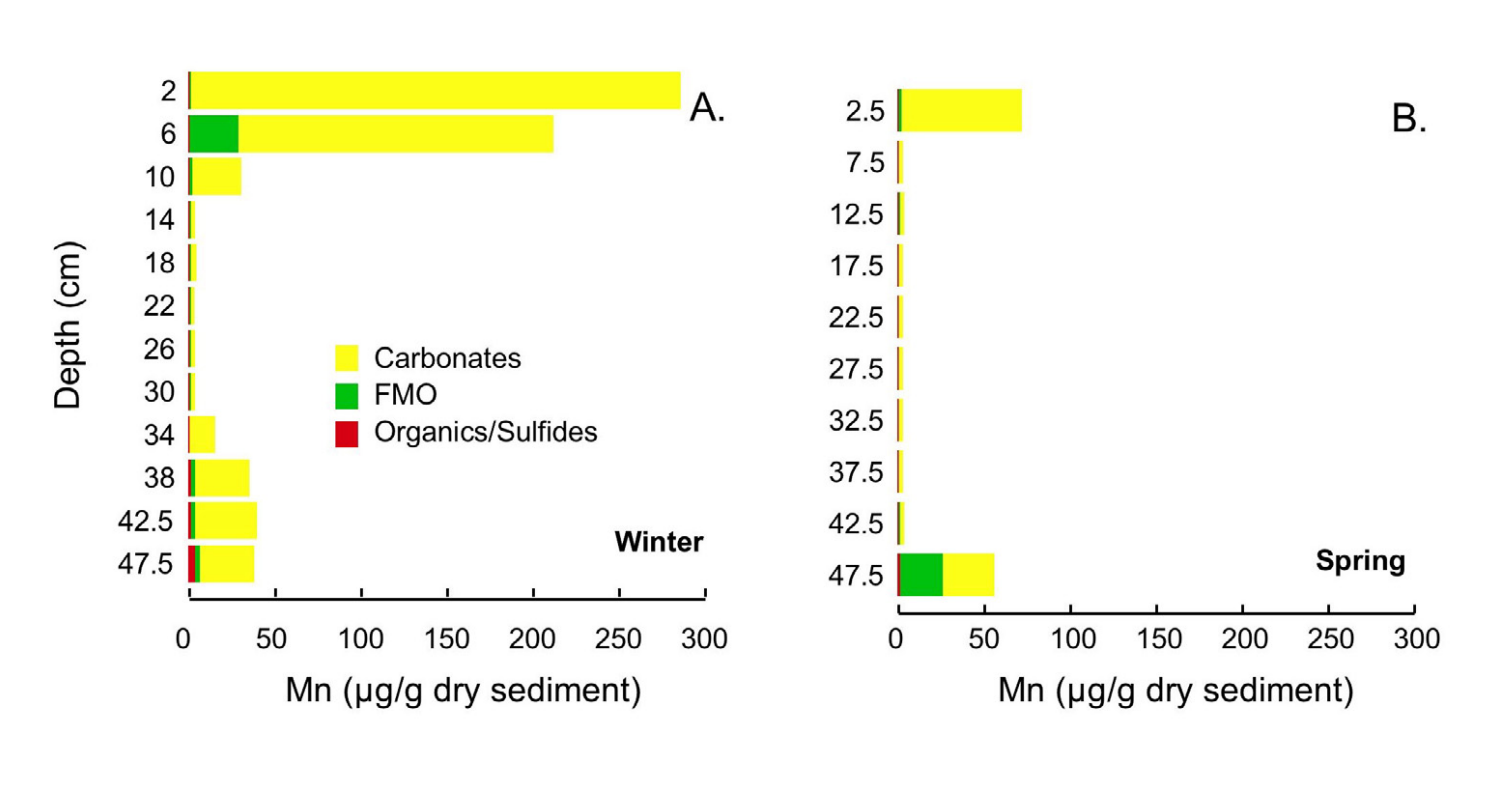
Mn, associated with three operationally-defined fractions (carbonates, FMO, and organics/sulfides) for cores collected in (A) winter (Mar 2002) and (B) spring (Jun 2002).

Extractable Mn concentrations exhibit a distinct depth dependence: in both cores, there is much more extractable Mn near the top and bottom of the core with concentrations in all three fractions near detection limits at intermediate depths (Fig 16). At nearly all depths, the majority of the extractable Mn is associated with the carbonate fraction. At a few depths a significant amount of Mn is extracted in the FMO fraction (e.g., up to 46% at bottom of spring core; up to 13% in 6 cm interval of winter core).

**Figure 17:**
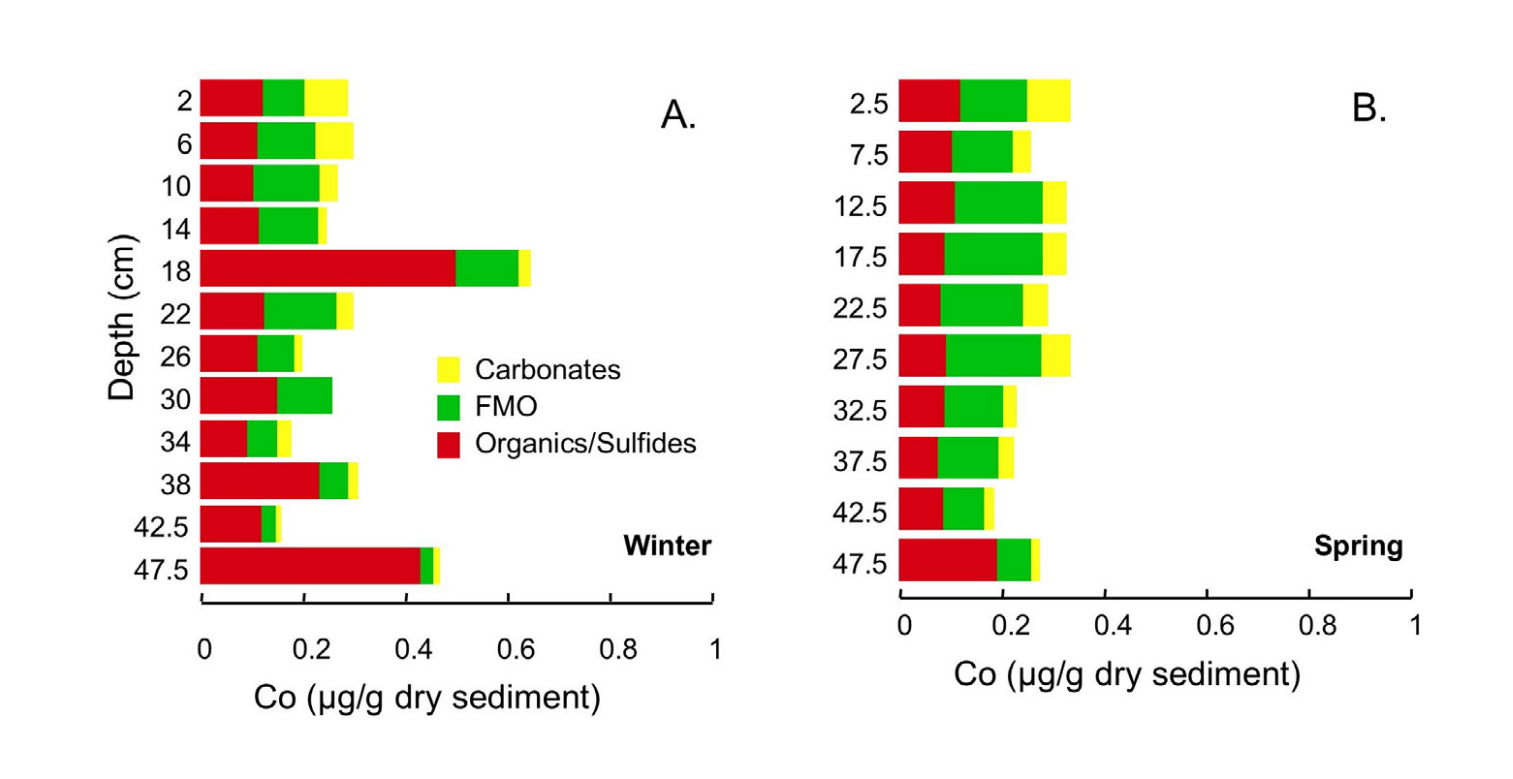
Co associated with three operationally-defined fractions (carbonates, FMO, and organics/sulfides) for cores collected in (A) winter (Mar 2002) and (B) spring (Jun 2002).

Carbonate, organic/sulfide and FMO extractable Co concentrations are low in both cores (<1 μg/g dry sediment; Fig. 17). There is little variation in total extractable Co concentration with depth in either core. In both cores, ~25–30% of the Co is associated with the carbonate fraction in the upper 5 cm of the core, declining to 5–10% of Co at the bottom of the core. There is an increase in the organic/sulfide extractable Co at the very bottom of both cores. Co associated with the FMO fraction accounts for 40–60% of the Co at most depths.

**Figure 18:**
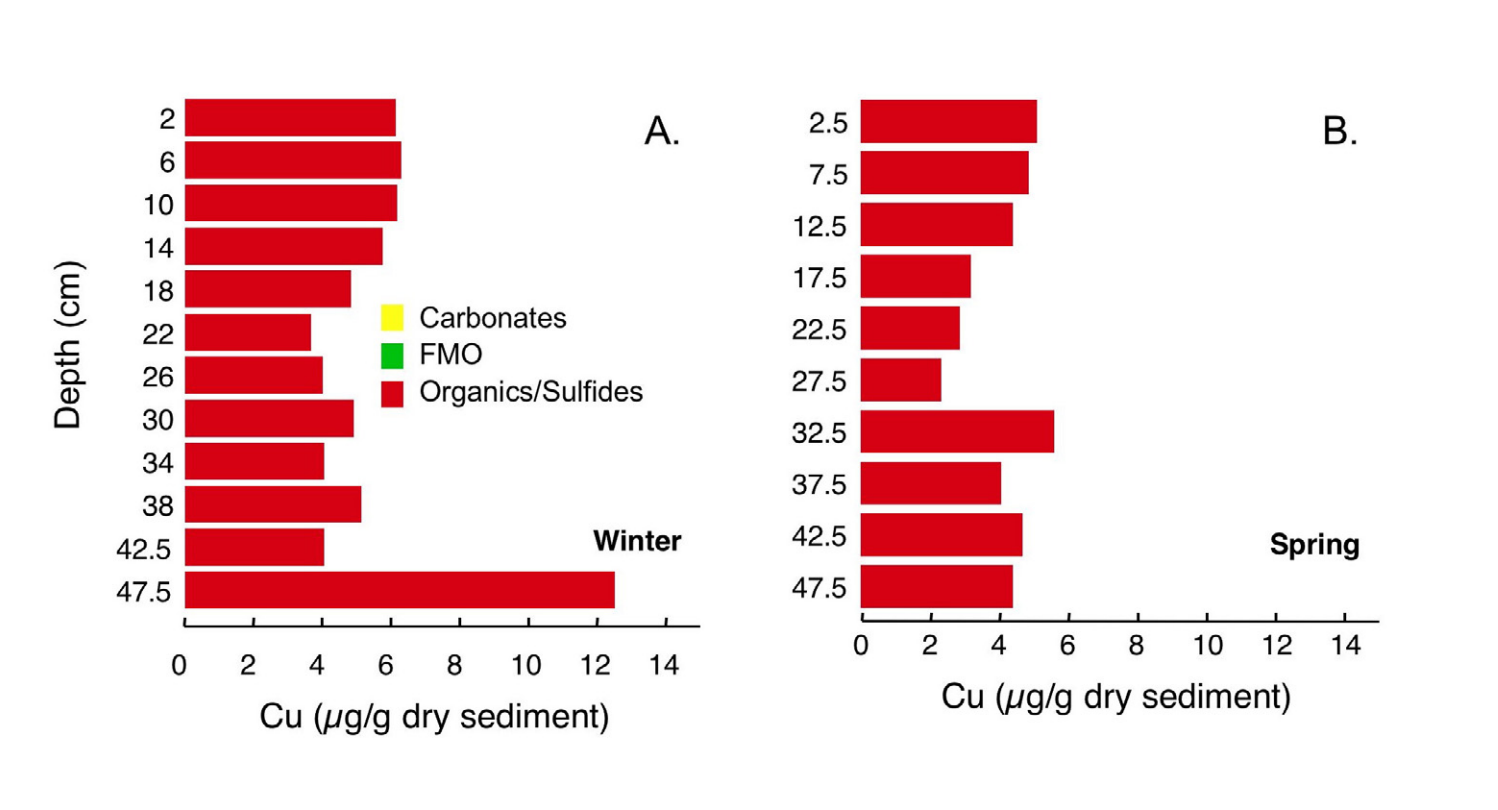
Cu associated with three operationally-defined fractions (carbonates, FMO, and organics/sulfides) for cores collected in (A) winter (Mar 2002) and (B) spring (Jun 2002).

Cu associated with the carbonate and FMO concentrations is below detection limits at all depths. Organic/sulfide extractable Cu concentrations are ~5 μg/g dry sediment and vary little with depth, with the exception of a large increase at the bottom of winter core (Fig. 18).

**Figure 19:**
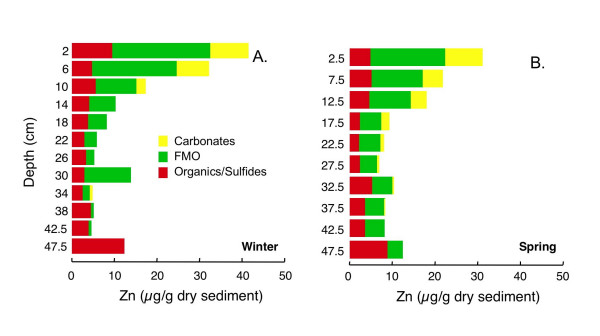
Zn associated with three operationally-defined fractions (carbonates, FMO, and organics/sulfides) for cores collected in (A) winter (Mar 2002) and (B) spring (Jun 2002).

Maximum total extractable Zn concentrations are close to 50 μg/g dry sediment in the winter core (Fig. 19A) and ~30 μg/g dry sediment in the spring core (Fig. 19B). In both cores, total extractable Zn declines with depth. In both winter and spring, ~5–10 μg/g dry sediment of Zn is associated with the organics/sulfides fraction with relatively little dependence on depth. In both winter and spring, ~10 μg/g dry sediment of Zn is extracted in the carbonates fraction in the upper most portion of the core. This decreases to <1 μg/g dry sediment by ~20 cm depth in spring and by ~15 cm depth in winter. In both cores, close to 20 μg/g dry sediment Zn is extracted with the FMO fraction at the top of the core. The concentration of Zn associated with the FMO fraction declines to <5 μg/g dry sediment by 50 cm depth in both spring and winter.

**Figure 20:**
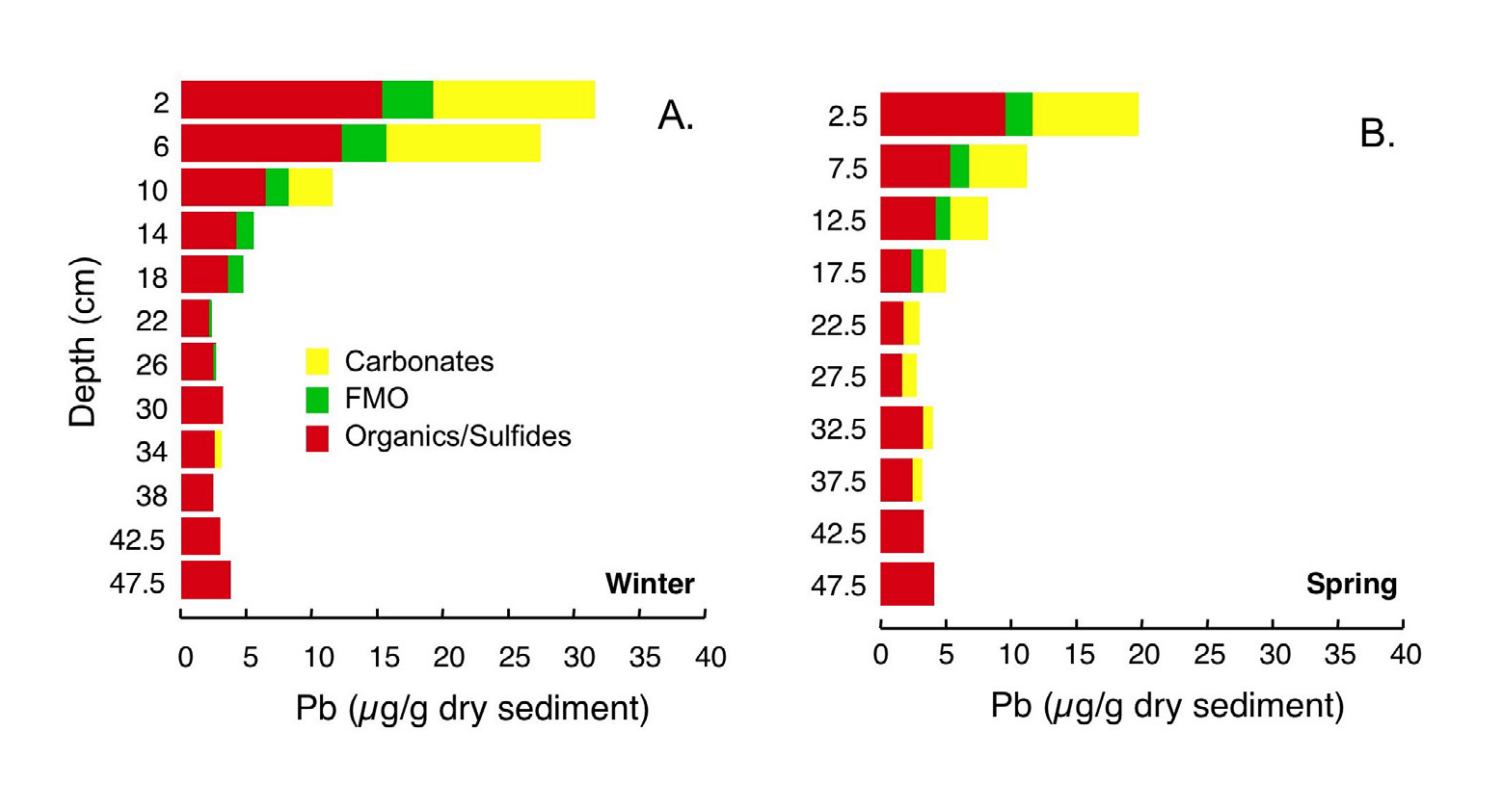
Pb associated with three operationally-defined fractions (carbonates, FMO, and organics/sulfides) for cores collected in (A) winter (Mar 2002) and (B) spring (Jun 2002).

Total extractable Pb concentrations, like Zn, are greatest in the uppermost portion of both cores, reaching ~30 μg/g dry sediment in winter (Fig. 20A) and ~20 μg/g dry sediment in spring (Fig. 20B), and decline with depth. At the top of both cores, ~45–48% of the Pb is associated with the organics/sulfides fraction, ~40% of the Pb with the carbonates fraction and the remaining 10–12% with the FMO fraction. The proportion of Pb associated with the carbonates and FMO fractions declines with depth in both cores, while the proportion associated with the organics/sulfides fraction increases with depth. The total concentration of Pb associated with each of the three fractions declines with depth.

## 5. Discussion

### 5.1 Redox Stratification

Sediment redox zonation can be recognized and delineated from measured pore water Fe(II), Fe(III), sulfate and sulfide depth-concentration profiles. Specifically, the presence of dissolved Fe(II) can be used to define a suboxic zone located just below the zone of oxygen penetration (oxic zone) and just above the sulfidic zone, which is defined by accumulation of dissolved sulfide [9]. In saltmarsh sediments, pore water redox stratification is strongly dependent on season, the presence or absence of vegetation and the activity of bioturbating organisms [e.g., [8,45]]. More compressed redox stratification (thinner zones, with the onset of the sulfidic zone nearer to the sediment surface) is favored by warmer temperatures, more dense vegetation and less bioturbation activity. Similarly, sulfur cycling and pore water composition in lake [5,11,13,16] and freshwater marsh [12] sediments and overlying waters have been shown to vary with season. This study demonstrates that redox stratification in the littoral sediments of a freshwater kettle lake also varies strongly with season.

Dissolved Fe(II) and sulfide pore water profiles suggest a gradual compression of redox stratification (i.e. suboxic and sulfidic zones occurring at shallower depth) from fall to summer. In fall, the suboxic zone stretches from ~1 cm above the SWI, where Fe(II) begins to accumulate in the pore water to at least 50 cm depth (Fig. 5A). Throughout this suboxic zone, sulfide levels are below detection limits (Fig. 6A). Upward migration of the suboxic zone in winter is apparent from the accumulation of Fe(II) at ~3–4 cm above the SWI and by declining Fe(II) concentrations below 20 cm depth (Fig. 5B). In spring, redox compression increases further, with Fe(II) accumulating ~8–10 cm above the SWI. Although peeper 2 remains suboxic throughout the measured depth profile, in peeper 1 a sulfidic zone appears below 20 cm depth (Fig. 5C, 6B). By summer, the sulfidic zone stretches from above the SWI to a depth of at least 50 cm in both peepers (Fig. 5D, 6C).

**Figure 21:**
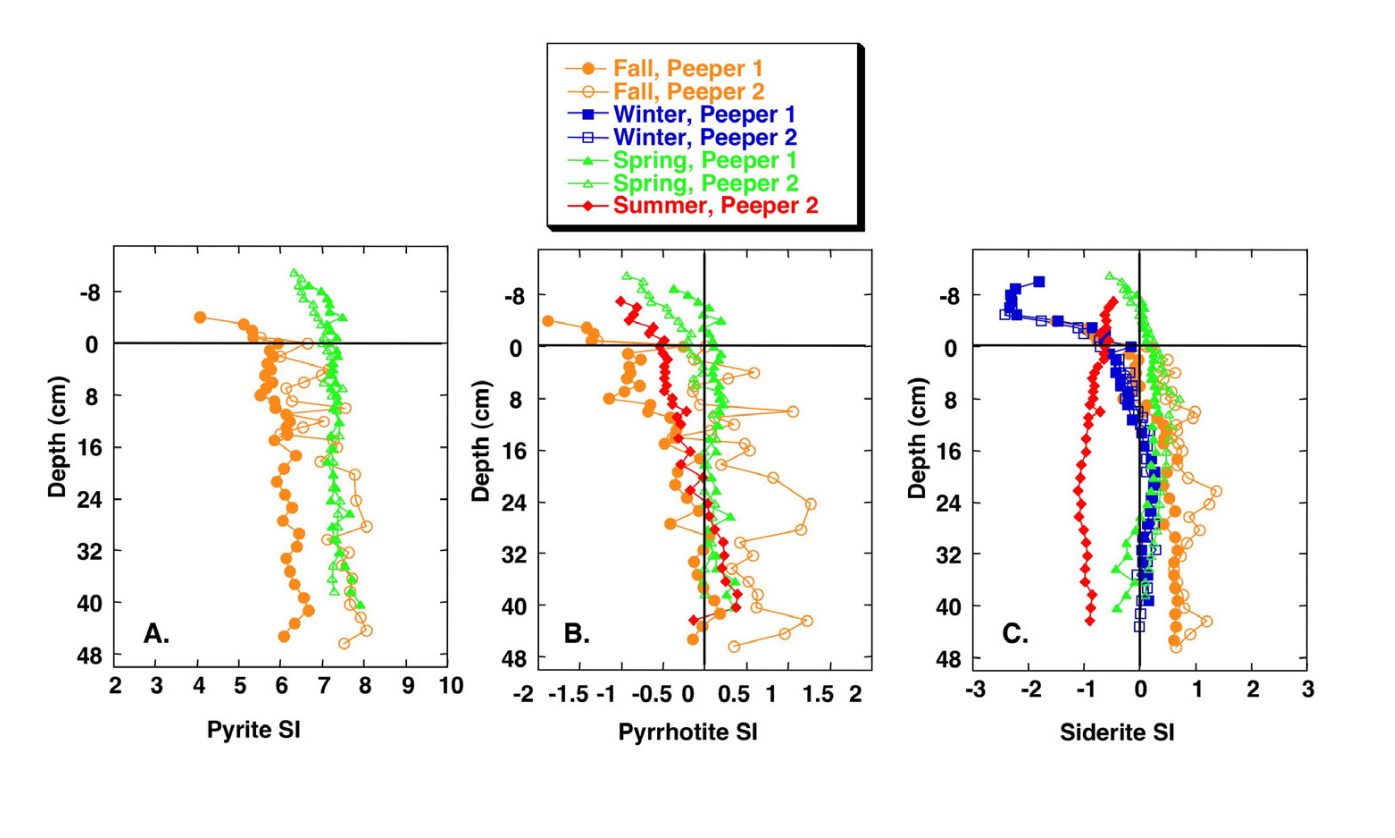
Saturation indices calculated using JCHESS for (A) pyrite, (B) pyrrhotite and (C) siderite, as a function of depth and season.

The decline in dissolved Fe(II) within the sulfidic zone is likely due to precipitation of amorphous and crystalline Fe sulfides, as has been observed in other freshwater sediments [3,46-50]. Calculations using the speciation code JCHESS [51] are used with pH and measured alkalinity and total dissolved Fe(II), SO_4_^-2 ^and ΣS^- ^to assess the saturation indices of several phases, including pyrite (FeS_2_), siderite (FeCO_3_) and pyrrhotite (FeS), as a function of sediment depth and season. Alkalinity is assumed to be equal to total dissolved bicarbonate as indicated by total inorganic carbon measurements completed during two seasons. The default JCHESS database is used with reactions and stability constants shown in Table 3. Pyrite is always strongly supersaturated, with the saturation index typically varying between 6.0 and 8.0 (Fig. 21A). Wersin et al. [52] also report high saturation indices for pyrite in lake sediments. Previous studies have shown that Fe monosulfides are close to saturation in lake sediments [e.g., [28,52]]. As seen in Fig. 21B, pyrrhotite is undersaturated above the SWI, but during fall, spring and summer is close to saturation in the sediments, consistent with removal of Fe(II) from the porewaters via precipitation of Fe monosulfides. It is also possible that siderite precipitates and removes Fe(II) from the porewaters. Except during summer, siderite is undersaturated above the SWI and is close to saturation in the sediments (Fig. 21C).

**Table 3:** Reactions and stability constants from JCHESS [51] used for speciation calculations.

**Reaction**	**Stability Constant**
Fe^+2^_(aq) _+ HS^-^_(aq) _+ 0.25H^+^_(aq) _+ SO_4_^-2^_(aq) _= FeS_2(s) _+ 1H_2_O pyrite	log K = 24.7
Fe^+2^_(aq) _+ HS^-^_(aq) _= FeS + H^+^_(aq) _pyrrhotite	log K = 3.72
Fe^+2^_(aq) _+ HCO_3_^-^_(aq) _= FeCO_3(s) _+ H^+^_(aq) _siderite	log K = 0.19

In most of the peepers, elevated Fe(III) concentrations are apparent in the pore waters just above the depths of maximum Fe(II) accumulation (Fig. 7). In agreement with seasonal changes in vertical redox stratification described above, the zone of Fe(III) accumulation migrates upwards from fall to summer. The presence of Fe(III) in the pore waters is consistent with oxidation of Fe(II) diffusing upward from the suboxic zone by oxygen (or other oxidants, such as nitrate or manganese) diffusing downward from the oxic zone [e.g. [6,7]] High levels of Fe(III) are most likely due to chelation with natural organic matter. In winter, cold, oxygenated waters may increase Fe(II) oxidation, increasing Fe(III) levels in the shallow surface waters, however, ice cover can also act as a barrier to oxygen [3].

Sulfate profiles were measured only during fall and spring (Fig. 8). Sulfate concentrations vary little with depth during fall, when the sediment pore waters are mostly suboxic. In contrast, during spring, as redox zones migrate upwards, sulfate concentrations decrease in the lower portion of the sediments. Diminishing sulfate levels are particularly apparent in peeper 1, the same peeper with decreased Fe(II) and accumulation of sulfide in the lowermost samples.

Seasonal changes in AEF are consistent with expectations from the inferred pore water redox stratification. AEF is thought to be composed primarily of readily-reducible amorphous Fe(III) (hydr)oxides [53,54]. During fall, when the pore waters point to suboxic conditions, maximum levels of AEF are observed. In summer, when the pore waters are sulfidic, AEF concentrations are at their lowest, presumably due to either chemically- or microbially-mediated reductive dissolution of Fe(III). During spring and winter, both redox compression and AEF levels are intermediate between the fall and summer extremes.

### 5.2 Dissolved Nutrients and Organic Matter Degradation Pathways

The oxidation of organic matter by oxygen releases protons and bicarbonate, as illustrated by the general reactions

"CH_2_O"_(aq) _+ O_2(aq) _= CO_2(aq) _+ H_2_O_(l) _    (1)

and

H_2_CO_3(aq) _= H^+^_(aq) _+ HCO_3_^-^_(aq) _    (2)

where "CH_2_O" represents labile organic matter. Protons are also released by O_2_-promoted reoxidation of reduced solutes, such as Mn(II), Fe(II) or H_2_S, for example,

4Fe^+2^_(aq) _+ O_2 _+ 8HCO_3_^-^_(aq) _+ 2H_2_O_(l) _= 4 Fe(OH)_3(s) _+ 8CO_2(aq) _    (3)

[55]. For this reason, pH profiles have been suggested as indicators of O_2_-penetration depths in saltmarsh sediments [45] and freshwater marsh sediments [2,12]. In fall and spring, when sediment redox stratification is least compressed, there is a decrease in pH near the top of the profile, consistent with oxidation of organic matter or reduced solutes by oxygen. In winter, the shallow water overlying the sediments lies beneath a frozen lake surface. The low pH values measured in both profiles during winter may reflect increased O_2_-promoted oxidation of organic matter and reduced solutes near the surface of these cold waters.

Measurements of total dissolved inorganic carbon in winter and spring demonstrate that the alkalinity profiles represent predominantly bicarbonate alkalinity. Bicarbonate is produced by anaerobic respiration of organic matter by dissimilatory manganese reduction, dissimilatory iron reduction and microbial sulfate reduction [e.g. [55]]. Bicarbonate can also be removed from the porewaters via precipitation of carbonate minerals. Therefore, the alkalinity profiles represent the net effect of organic matter degradation and carbonate precipitation. Throughout most of the profiles, alkalinity is nearly constant with depth (Fig. 2). This probably reflects saturation with respect to a carbonate phase, such as siderite (Fig. 21C), or more likely calcite (CaCO_3_) or rhodochrosite (MnCO_3_), but without dissolved Ca and Mn data this question cannot be satisfactorily resolved. The lower alkalinity values observed at the top of the fall and winter profiles (Fig. 2A,B) likely reflect the less compressed redox stratification during these seasons and are consistent with organic matter degradation coupled to oxygen as the terminal electron acceptor at the top of these profiles. The rapid increase in alkalinity with depth indicates the onset of anaerobic organic matter degradation well above the SWI.

Dissolved phosphate is another potential tracer of organic matter degradation, because it is released during mineralization of natural organic matter. However, because phosphate associates strongly with Fe(III) oxides [e.g. [14]], dissolved phosphate can also be released to solution by reductive dissolution of Fe oxides [e.g. [48,49,56-58]]. Dissolved phosphate, frequently a limiting nutrient in freshwaters, can also be efficiently removed from solution by macrophytes and other organisms [59,60]. The dissolved phosphate profiles thus reflect the sum of these processes. Low levels of dissolved phosphate during summer (Fig. 3C) most likely indicate efficient uptake of phosphate by aquatic organisms and not a lack of organic matter degradation. The large peak in phosphate observed in one of the peepers during fall (Fig. 3A) probably does reflect organic matter mineralization. The high phosphate levels above the SWI in one of the summer peepers (Fig. 3C) can be attributed to a combination of organic matter mineralization and possibly release of phosphate from the reductive dissolution of Fe(III) oxides (see discussion of solid phase Fe speciation below).

Like dissolved phosphate, dissolved ammonium is produced by mineralization of organic matter and can be consumed by aquatic organisms. In oxidized sediments, dissolved ammonium can also be removed via nitrification. In fall, a peak in ammonium occurs just above the SWI, coincident with a peak in dissolved phosphate (Fig. 4A). This likely reflects organic matter mineralization. The much higher levels of ammonium observed in the other peeper may be due to spatial heterogeneity with respect to vegetation and uptake of ammonium by macrophytes. Similarly, differences in levels of ammonium observed in the two sets of peeper data collected in spring (Fig. 4B) could be caused by patchiness of macrophyte distribution and ammonium uptake. Declining levels of dissolved ammonium with depth during spring, and low levels of ammonium beneath the SWI in summer, again point to efficient uptake of this nutrient by macrophytes. Although nitrification is unlikely to occur under the relatively reducing conditions of the "bulk" sediments, it is possible that nitrification plays an important role in nitrogen transformations near macrophyte roots or irrigated burrows, where oxygen may be directly introduced into the sediments at depth [61-64].

Without measured reaction rates for nitrogen transformations, microbial sulfate reduction and dissimilatory or chemical iron or manganese reduction, it is not possible to make a quantitative assessment of natural organic matter degradation pathways in these sediments, as has been done for other lakes [7]. Furthermore, no data regarding transport of solutes by advection, bioirrigation or root leakage or of solids by bioirrigation [65] are available for these sediments. Nonetheless, the data do allow some important qualitative observations.

Accumulation of Fe(II) or sulfide above the SWI suggests that organic matter degradation coupled to oxygen or nitrate as terminal electron acceptor will be limited to a zone well above the SWI, or to patches in the sediment containing oxygen introduced by macrophyte roots [e.g. [66-68]] or bioirrigating macrofauna [e.g [6,45]]. Tessier extractions suggest that Mn is primarily associated with carbonates (Fig. 16). Thus, organic matter mineralization coupled to Mn(IV) reduction is unlikely to be a dominant pathway, unless manganese is cycled so quickly that Mn(IV) cannot accumulate in the sediments [3]. The large quantity of AEF, especially in the upper portion of the sediment column during fall, winter and spring, (Figs. 10, 11, 12), together with the accumulation of dissolved Fe(II), implies that dissimilatory iron reduction is an important pathway for organic matter mineralization. This is consistent with quantitative estimates of organic matter oxidation in the upper 18 cm of deep lacustrine sediments from Lake Michigan, which point to the importance of microbial iron reduction (44%) with lesser sulfate reduction (19%) or oxidation by oxygen (37%) and negligible coupling to manganese reduction [7]. In summer, organic matter degradation via microbial sulfate reduction probably becomes much more significant in these shallow sediments. Very high rates of microbial sulfate reduction have been measured in other freshwater sediments, in spite of relatively low concentrations of sulfate [69-72]. Thus, the small quantity of dissolved sulfate does not preclude microbial sulfate reduction as a significant organic matter degradation pathway. AEF, while not entirely absent, is present in much lower quantities in the summer cores than during other seasons. If the remaining AEF is comprised of the most refractory Fe, microbial sulfate reduction will more easily outcompete dissimilatory iron reduction [3,73]. Furthermore, studies of saltmarsh sediments have demonstrated that microbial sulfate reduction can produce quantities of sulfide sufficient to allow sulfate reducers to outcompete iron reducers [8]. The mechanism of competition is hypothesized to be removal of labile Fe(III) oxides via chemical reduction (by sulfide), amplified by macrofaunally-mediate solute transport. Similar processes could occur in these sediments, but without measurements of microbial sulfate reduction rates, iron-reducing bacteria populations or activities or bioirrigation intensities, this cannot be substantiated.

### 5.3 Comparison of Extraction Schemes: Fe Speciation

The Kostka and Luther extractions suggest that Fe occurs mostly as amorphous Fe(III) (hydr)oxides (AEF), with a much smaller pool of Fe in acid volatile sulfides (HEF) present during all seasons and at nearly all sediment depths (Figs. 10, 11, 12, 13). AEF varies considerably with season and with depth in these sediments, with this variability typically greater than what is observed for replicate cores. Much more AEF is present, especially in the uppermost portion of the cores, during fall and winter than in spring and summer. This is in agreement with seasonal variations in redox geochemistry inferred from the pore waters, as described above, and suggests that AEF is a pool of Fe that undergoes a seasonal cycle of reductive dissolution and oxidative precipitation. In all seasons, AEF decreases significantly with depth. This is consistent with the expected spatial sequence of redox reactions, sometimes termed a redox loop, which concentrates Fe and Mn oxides in near-surface sediments [e.g. [3,6]].

Operationally-defined Fe extractions based on the Tessier method (Fig. 14) give a very different picture of Fe distribution in these sediments compared to the Kostka and Luther extractions (Fig. 10, 11, 12, 13). The Tessier extraction produces more Fe total (sum of the five extraction steps) then the two steps of Kosta and Luther. This is not surprising, as the Kostka and Luther method should only target amorphous Fe(III) oxides and AVS, whereas the 5-step Tessier extraction should remove all Fe from the sediments. However, in contrast to the Kostka and Luther results, the Tessier extraction indicates that most of the Fe is associated not with FMO, but rather with the oxidizable organics/sulfides fraction. Much more organics/sulfides associated Fe is extracted then by either, or indeed by the sum, of the two Kostka and Luther steps. The depth distribution of the organics/sulfides fraction is unlike that of AEF or HEF, instead closely resembling the depth distribution of LOI (Fig. 9). This suggests that much of the Fe extracted in this fraction is associated with organic matter, rather than sulfides.

The Tessier FMO fraction, which should correspond to the AEF fraction of Kostka and Luther, accounts for the second largest pool of Fe extracted with the Tessier method. Interestingly, there is a relatively good correlation between the Tessier FMO fraction and the Kostka and Luther HEF fraction (R^2 ^of 0.88 in winter and R^2 ^of 0.55 in spring). Furthermore, in winter, nearly identical total quantities of Fe are extracted in the FMO and HEF steps. During spring, the quantities of iron extracted by the two methods are similar in the lower portion of the core, but much more Fe is extracted as FMO than as HEF in the upper 30 cm. The correlation between FMO and HEF is unexpected, as these two extraction steps are supposed to target quite different sediment fractions. Possible explanations include: (1) Kostka and Luther HEF step actually extracts Fe(III) oxides, (2) Tessier FMO step actually extracts acid volatile sulfides, or (3) HEF and FMO both extract Fe from carbonates or another non-target phase.

The first explanation is unlikely, because the HEF extractions are dominated by Fe(II), with almost no Fe(III). It could perhaps be argued that HEF actually represents Fe(III) originating from Fe(III) oxides reductively dissolved in the ascorbate extraction, which is reduced, released and adsorbed onto remaining sediment particles and then released as Fe(II) in the HCl extraction step. If this is the case, it is not clear why only the fraction targeted by the Tessier FMO would be adsorbed and released as HEF, while a much larger quantity of Fe is released in the Kostka and Luther ascorbate extraction. In fact, a similar correlation between Tessier FMO and Kostka and Luther HEF does not occur in freshwater marsh sediments [12], so this explanation seems unlikely.

The second possibility is that the Tessier FMO technique does not release Fe from oxides, but rather releases Fe associated with acid volatile sulfides. This is plausible, especially given that freeze-drying in air may oxidize and redistribute acid volatile sulfides [74,75]. Other workers have demonstrated that the hydroxylamine HCl used to extract FMO can partially release amorphous sulfides [76,77]. If this is the case, then another question arises: given the large quantity of AEF, why isn't more FMO extracted? Specifically, why is it not equal to the quantity of AVS plus the AEF? In fact, AEF profiles from the Kostka and Luther technique are similar in magnitude and depth-distribution to the oxidizable Fe released by the Tessier technique. Thus, AEF must either be composed largely of organic-bound Fe, or Fe oxides must be released in the organics/sulfides of the Tessier method. Given the excellent agreement between seasonal changes in AEF and redox conditions inferred from the pore waters, it does not seem plausible that the AEF is comprised primarily of organic-bound Fe. A more likely explanation is that Fe oxides are at least partially released in the organics/sulfide step of the Tessier method. This could reflect an insufficient quantity of hydroxylamine HCl to extract all of the Fe in these sediments, as has been demonstrated for more Fe-rich sediments [78-80].

A third possibility is that neither Tessier FMO, nor Kostka and Luther HEF extract the targeted phases. For example, both might actually remove Fe(II) from carbonates. In anoxic lake sediments, freeze-drying has been shown to increase the quantity of Fe extracted in a reducible phase, with most of the "excess" reducible Fe mobilized from a combined exchangeables/carbonates fraction [75]. This was attributed to oxidation of poorly crystalline siderite [75]. This explanation, however, would require carbonates to escape extraction in both the Tessier carbonate extraction phase and the ascorbate extraction of Kostka and Luther. This discussion illustrates the inherent difficulties in interpretation of data from operationally-defined extraction techniques (see also reviews of Refs. [81] and [82]).

In summary, the most likely scenario appears to be that the Kostka and Luther AEF removes a mixture of Fe associated with readily reducible oxides and organics, while the Tessier organics/oxidizable fraction removes a mixture of Fe associated with readily reducible oxides, organics and disulfides. Thus, the general depth-distribution of organic/sulfide associated Fe is similar to LOI profiles, except that Fe is enriched at the top of the organic/sulfide cores compared to the LOI profiles. This is not surprising, as redox reactions should serve to concentrate Fe(III) oxides into the uppermost portion of the sediments. At the bottom of the cores, larger Fe concentrations extracted in the Tessier organics/sulfides compared to Fe extracted by Kostka and Luther may indicate the presence of Fe(II) disulfides (e.g. pyrite) at depth. The Tessier organics/sulfides step is likely to remove a significant quantity of Fe disulfides [76], whereas the Kostka and Luther technique only removes Fe monosulfides. Other studies have shown progressive aging of Fe monosulfides to Fe disulfides over time [83], so it is not surprising that larger quantities of Fe disulfides would be present deeper in the sediment.

### 5.4 Trace Metal Distribution

The significant seasonal variations in redox stratification of these sediments clearly influences Fe cycling. Therefore, significant seasonal variations in trace metal speciation should be expected. Reductive dissolution of FMO should mobilize associated metals, which, like Fe(II) or Mn(II) may diffuse upwards into a more oxidizing zone, where they could be reincorporated into fresh FMO. Mobilized chalcophiles diffusing downwards to more reduced sediments will likely become associated with sulfides, while lithophiles such as Mn or Cr may be incorporated into carbonate phases. Similarly, when redox stratification becomes less compressed and reducing sediments become more oxidized, degradation of sulfide phases may release and mobilize associated metals. These metals could diffuse downwards and become incorporated in other sulfides, or might diffuse upwards and associated with oxides. Depending on the geochemistry of a particular element, seasonal precipitation and dissolution of FMO and AVS could lead to significant changes in partitioning among the pore waters and various sediment constituents. In this study, trace element partitioning among operationally defined solid phases was assessed during winter and spring only. Kostka and Luther extractions and pore water sampling conducted during all seasons suggests that comparisons of winter and spring only will underestimate the potential magnitude of change throughout the entire year. Nonetheless, comparisons between these two seasons and possible implications for the measured trace elements are discussed below.

#### Chromium

According to the Goldschmidt classification, Cr is a lithophile [84]. It is frequently associated with organic matter [85,86] or FMO [3,17,34]. Pyrite and sulfides are not likely to be important sinks for Cr [27,29,86]. Freeze-drying may increase the proportion of Cr in the exchangeable fraction at the expense of carbonate and FMO associated Cr [87].

Nearly all of the Cr in both seasons is associated with the reducible FMO fraction (Fig. 15). Cr has been shown to have an affinity for Mn oxides [3]. However, the large increase in FMO-associated Cr between 5–15 cm in winter does not correspond to a similar increase in any other measured metal profile, including Mn, although there is a small elevation in FMO-associated Mn centered at ~6 cm (Fig. 16). Boyle [88] used a quantitative reactive-transport model to demonstrate that under low sediment accumulation rates metals such as Cu, Pb and Zn would be elevated in the solid phase of a freshwater sediment just below Fe oxide peaks, due to sediment advection. It is possible that such a mechanism operates here, but it seems unlikely that this would result in the very large observed enrichments of Cr. Furthermore, in spring, no such enrichment is observed.

#### Manganese

Like Cr, Mn is a lithophile element. In suboxic or anoxic sediments, Mn is most often found in association with carbonate phases [3,86,89], although some Mn may adsorb on AVS [28] or may become incorporated into sulfides at high degrees of pyritization [27,29]. Mn is not typically associated with organic matter [3]. Under oxidizing conditions, Mn(IV) oxides form and may be an important phase for incorporation of metals such as Co, Zn and Cr [3,15]. Some studies have found that freeze-drying mobilizes Mn from exchangeable and carbonates fractions into the FMO pool [74,87], although other studies show little change in partitioning as a consequence or freeze-drying [75] or sediment aeration [90]. In this study, very little Mn is extracted with the FMO pool (Fig. 16), so mobilization into this fraction during freeze-drying seems unlikely.

Nearly all of the extracted Mn during both seasons is associated with carbonates (Fig. 16). This is consistent with reductive dissolution of Mn(IV) oxides above the SWI, as has been seen in previous studies of lake sediments [3,16,17], with subsequent precipitation of reduced Mn(II) to form either rhodochrosite or a solid solution with calcite. Profiles of carbonate associated Mn are similar to the LOI profiles, suggesting that the texturally-distinct low organic matter layer observed in the middle of the cores is not a marl, but more likely a clay-rich layer, depleted in both carbonates and organic matter.

In winter, depth profiles of carbonate associated Mn correlate well with Zn (R^2 ^= 0.94), Pb (R^2 ^= 0.91) and Co (R^2 ^= 0.84) extracted as carbonates. These correlations are not as strong in spring, when less carbonate associated Mn is present in the sediments. This decrease in carbonate-Mn in spring is unexpected, as the higher organic matter degradation rates in spring should produce more bicarbonate. Thus, it seems most plausible that the observed differences are due to sediment heterogeneity, rather than a seasonal effect. However, another possible explanation is that the carbonate-extractable Mn, Pb, Zn and Co in winter originate, at least in part, from a monosulfide phase oxidized during sample pretreatment, mobilizing these elements into the carbonate phase. Such effects have not been previously reported for Mn, although this is a distinct possibility for Pb and Zn [74,75,87].

The decrease in Mn associated with the FMO phase near the top of the profile from winter to spring may likewise be due to sediment heterogeneity, or, it could indicate that a small quantity of Mn oxides present during winter are reductively dissolved in the winter to spring transition. The slight elevation of Mn in the FMO phase at the bottom of the spring core is also unexpected, and could indicate a local patch of more oxidized sediment, due to root pumping of oxygen, for example.

#### Cobalt

Co may adsorb on sulfides, and pyrite or CoS are likely to be important sinks [27-29]. Organics are probably not an important reservoir for Co [28]. In more oxidized sediments or waters, oxides may be a significant sink for Co [3,17]. Balistrieri et al. [17] measured similar seasonal changes in Co and Mn concentrations in lake column waters. They suggested that Co associated with Mn oxides is released during reductive dissolution of the Mn oxides and is then partially scavenged by Fe oxide particulates.

Co is associated with organics/sulfides, FMO, and to a lesser extent, with carbonates, with very similar profiles in winter and spring (Fig. 17). In both seasons, there is a decrease in FMO and carbonates associated Co with depth, with an increase in organics/sulfides associated Co, especially at the bottom of each core. The organics/sulfides associated Co is likely associated with sulfides, rather than organic matter. The increase at the bottom of the core is consistent with formation of pyrite with associated Co at depth. However, the depth profile of organics/sulfides associated Co does not correlate well with organics/sulfides associated Fe. This is likely because organic/sulfide extractable Fe is present in multiple mono- and di-sulfide phases and possibly oxides (see above), all of which may not contain Co.

As discussed above, in winter, there is a linear correlation between carbonates associated Co and Mn profiles (R^2 ^= .84). There is also a strong correlation between profiles of carbonate-Co and carbonate associated Zn (R^2 ^= 0.92) and Pb (R^2 ^= 0.91). As discussed above, this may indicate that all of these elements are present in the same carbonate phase, or, alternatively, that they are all mobilized from the same sulfide phase during freeze-drying.

In spring, correlations between carbonate associated Co and Pb (R^2 ^= 0.58) or Zn (R^2 ^= 0.52) are weaker than in winter. Furthermore, there is not a good correlation between carbonate-associated Co and Mn (R^2 ^= 0.30) in spring. There is a somewhat better correlation with carbonate-extracted Fe (R^2 ^= 0.57). This might indicate that Co switches from an associated with predominantly rhodochrosite in winter to greater association with siderite in spring.

Co extracted with FMO is likely associated with Fe oxides, rather than Mn oxides, consistent with an observed correlation with FMO-extracted Fe (R^2 ^=.70) and no correlation with FMO-extracted Mn (R^2 ^= .03).

#### Copper

Copper is a chalcophile element with a very strong affinity for organic matter and sulfides [91]. In reducing sediments, Cu may form discrete Cu sulfide minerals [17,27,34] or may be associated with pyrite [29,34,86] or FeS [17]. Copper is frequently complexed to organic matter [e.g. [34,85,92,93]]. It is sometimes associated with FMO and may form ternary complexes with organic matter bound to FMO [18]. Thus, reductive dissolution of FMO can release Cu-organic complexes that subsequently associate with sulfides [34].

Freeze-drying of anoxic sediments has been reported to mobilize Cu into the exchangeable or carbonates fractions [74,75] or into the FMO fraction [75,87,94], probably due to oxidation of AVS. Buykx et al. [90] observed that aeration had little influence on operationally-defined Cu partitioning, which they attributed to Cu being associated primarily with organics in their samples. In this study, all of the non-residual Cu is extracted with the oxidizable fraction and was clearly not mobilized into the carbonate or FMO fractions during sample pretreatment (Fig. 18).

Oxidizable Cu concentrations are similar in winter and spring. Both Cu profiles show relatively little variation with depth, except that in winter there is a large increase in extracted Cu at the bottom of the core. This could indicate an increase in organic matter with associated Cu: the LOI profile for this winter core also increases at the bottom of the core. However, a similar increase in LOI in the spring core is not accompanied by an increase in extractable Cu. Thus, the increase in Cu at the bottom of the core in winter is more likely due to an increase in Cu associated with disulfides, as inferred for Co.

#### Zinc

Zn is a chalcophile and likely forms distinct sulfide phases, rather than being incorporated into pyrite [27-29,34]. Under oxidizing conditions, Zn probably associates mostly with FMO, while under anoxic conditions, it is found in association with sulfides and carbonates [3,95]. Zn also has a strong affinity for organic matter [34,93,96]. Zn cycling in lake columns may be linked to changes in biological activity and silicate particulates [17]. A study of seasonal changes in Zn partitioning in wetlands sediments demonstrated that Zn speciation responds rapidly to changes in sediment redox [95]. Interestingly, the authors also observed that Zn partitioning inferred from sequential extractions on wet sediment samples was generally in good agreement with XAFS data.

Freeze-drying has been reported either to have little effect on Zn partitioning [94] or to mobilize Zn from the organics/sulfides pool to the exchangeable, carbonates or FMO fractions [74,75,87], presumably due to oxidation of a Zn sulfide phase. Similarly, Bukyx et al. [90] report that aeration of reduced sediment results in mobilization of Zn from a sulfide phase to a carbonate phase. In another experimental study, Carbonaro et al. [32] showed that oxidation of Zn-spiked AVS resulted in rapid mobilization of Zn into overlying water, followed by slow incorporation of Zn into an FMO phase.

The largest proportion of Zn in both winter and summer is associated with FMO (Fig. 19), which could indicate oxidation of a monosulfide Zn-bearing phase during freeze-drying. This would result in an overestimation of FMO, and possibly carbonate, associated Zn. There is somewhat less Zn in the FMO phase during spring, which could indicate reductive dissolution of Zn-bearing FMO. However, no increase in Zn associated with a sulfide phase is observed in winter, so differences between Zn in winter and spring could simply be due to sediment heterogeneity.

As discussed above, Zn extracted in the carbonate phase during winter correlates strongly with Pb (R^2 ^= 0.99), Mn (R^2 ^= 0.94) and Co (R^2 ^= 0.91), suggesting that all of these elements are either associated with the same carbonate phase, or possibly with the same sulfide phase oxidized during freeze-drying. A strong correlation between the chalophile elements Zn and Pb extracted with carbonates is also present in spring (R^2 ^= 0.98), but correlations with the Co (R^2 ^= 0.52) and Mn (R^2 ^= 0.49) are weaker. This suggests that Pb and Zn coexist in the same phase, either a carbonate phase, or possibly a sulfide phase oxidized during freeze-drying, during both winter and summer.

The largest pool of Zn, FMO associated, correlates strongly with Pb (R^2 ^= 0.85 in winter, 0.90 in spring) and more weakly with Fe (R^2 ^= 0.57 in winter, 0.54 in summer). Pb and Zn extracted in the FMO step may be associated with Fe oxides, although given the good correlation between Fe extracted as FMO and Fe extracted as HEF in the Kostka and Luther extraction, contributions of Fe, Pb and Zn from sulfide phases oxidized during freeze-drying may be significant. The sharp increase in organics/sulfides extractable Zn at the bottom of both cores is probably associated with a disulfide phase, as for Fe, Co and Cu.

#### Lead

Lead, like Cu, is a chalcophile and chelates strongly with organics [96]. It also sorbs strongly on FMO [14] and may be entrained in freshly precipitated Fe oxides [97]. Like Cu, Pb may form ternary complexes with organic matter bound to Fe oxides [18,98]. Enrichment of Pb near the surface of lake sediments has been attributed to a combination of complexation with organics and FMO [93]. Lead is not incorporated into pyrite, but can form distinct sulfide phases [27-29]. Oxidation of Pb-spiked AVS has been shown to result in release of Pb that is quickly sorbed to FMO [32]. Freeze-drying may shift Pb associated with oxidizable phases to exchangeable, carbonates or FMO fractions [75,94]. However, Buykx et al. [90] found little influence of sediment aeration on operationally-defined Pb partitioning, which they attributed to binding of Pb primarily to organics.

The majority of Pb is extracted with the organic/sulfides fraction (Fig. 20). However, a significant proportion of Pb is extracted in the carbonates fraction. This fraction of Pb correlates strongly with Zn (R^2 ^= 0.99 in winter, 0.98 in spring), Co (R^2 ^= 0.91 in winter, 0.58 in spring) and Mn (R^2 ^= 0.91 in winter, 0.51 in spring). This fraction of the Pb might be present in rhodochrosite (winter) or siderite (spring), or may have been mobilized from a sulfide phase during freeze-drying.

The small quantity of Pb extracted with FMO decreases with depth and correlates with Fe (R^2 ^= 0.75) in spring. There is less FMO associated Pb in spring than in winter, which might indicate loss of Pb to a more reduced pool in the winter to spring transition, or this could be simply due to sediment heterogeneity.

The largest pool of Pb is associated with organics/sulfides that were apparently not oxidized and mobilized into other fractions during the freeze-drying. This Pb is most likely associated with organics together with mono- and disulfide minerals. The slight increase in Pb in this fraction at the bottom of both cores, in particular, points to association with a disulfide phase.

## 6. Conclusion

This study demonstrates that organic-rich, littoral lake sediments undergo significant seasonal variations in pore water and sediment redox stratification. Specifically, redox stratification becomes progressively more compressed, as defined by a shallower, thinner suboxic pore water zone in the upper 50 cm, from fall to summer. Without rate data, it is not possible to quantify the contribution of different redox pathways to organic matter oxidation in these sediments. However, pore water and solid phase profiles suggest an important role for ferric iron and sulfate reduction, with much less organic matter degradation in the upper 50 cm of sediment coupled to oxygen, nitrate or manganese reduction.

Operationally-defined sequential extraction data and pore water redox stratification data are consistent with a model of dynamic cycling of solid phase Fe in agreement with the seasonal changes observed in pore water redox stratification. Seasonal changes in the distribution of redox sensitive solid phases, including FMO and sulfides, are likely to significantly influence trace metal speciation. Fe, Cu, Pb and Co, which associate strongly with the organics/sulfides fraction, will be most influenced by cycles of AVS and pyrite formation and subsequent oxidation and degradation. Significant quantities of Cr, Cu and Zn are extracted with the FMO phase, and will thus be influenced by cycles of reductive dissolution and oxidative precipitation of Fe and Mn oxides. Mn is mostly present in the carbonates phase, probably because reactive Mn oxides are reduced in the water column.

Lastly, although operationally-defined sequential extraction techniques may have the potential to provide useful insights into seasonal changes in trace element cycling, results must be interpreted with some caution due to inherent problems with such methods, including lack of selectivity and the potential for changes in metal distribution during sample pretreatment. Application of two or more methods to the same sediments may provide constructive insights regarding metal speciation.
